# Supplementary figures and images for: SARS-CoV-2 NSP13 interacts with TEAD to suppress Hippo-YAP signaling
Source: eLife. 2025 Sep 23;13:RP100248. doi: 10.7554/eLife.100248 (PMC12456957; doi:10.7554/eLife.100248)

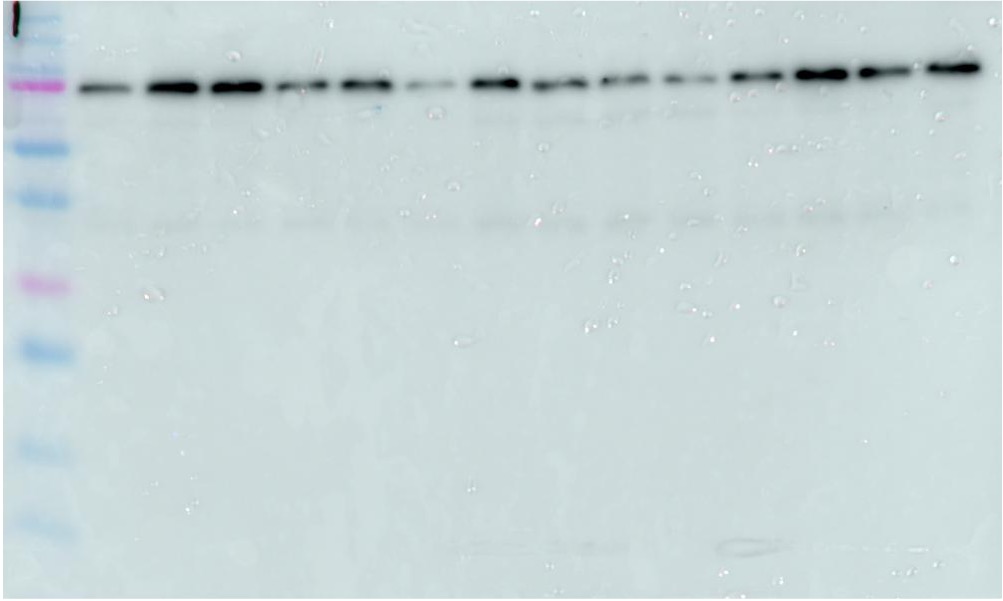

Supplement: Figure 2—source data 1. [file elife-100248-fig2-data1.zip › Flag-YAP WT.jpg]

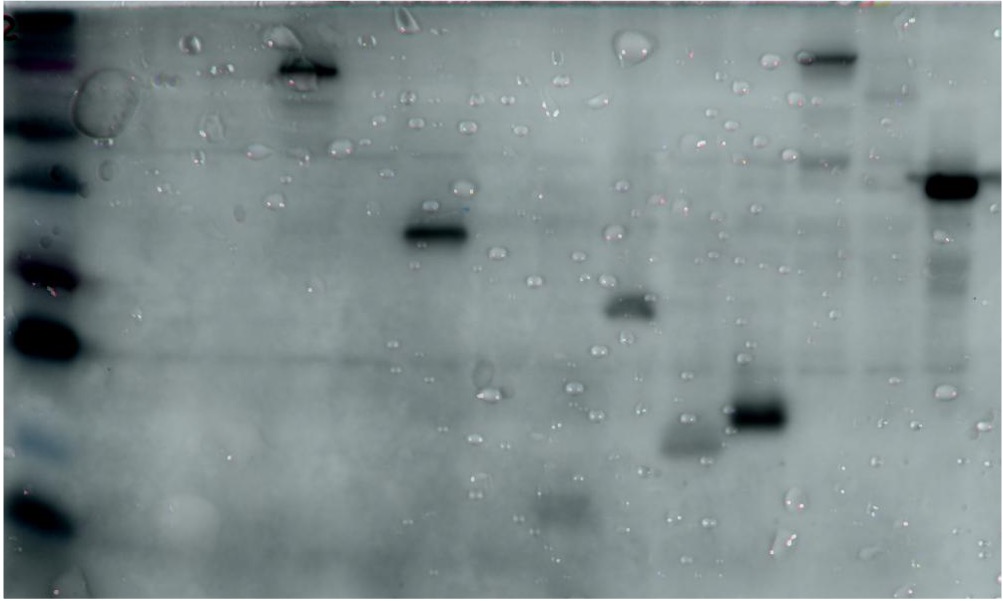

Supplement: Figure 2—source data 1. [file elife-100248-fig2-data1.zip › Strep-NSPs.jpg]

Figure 2A

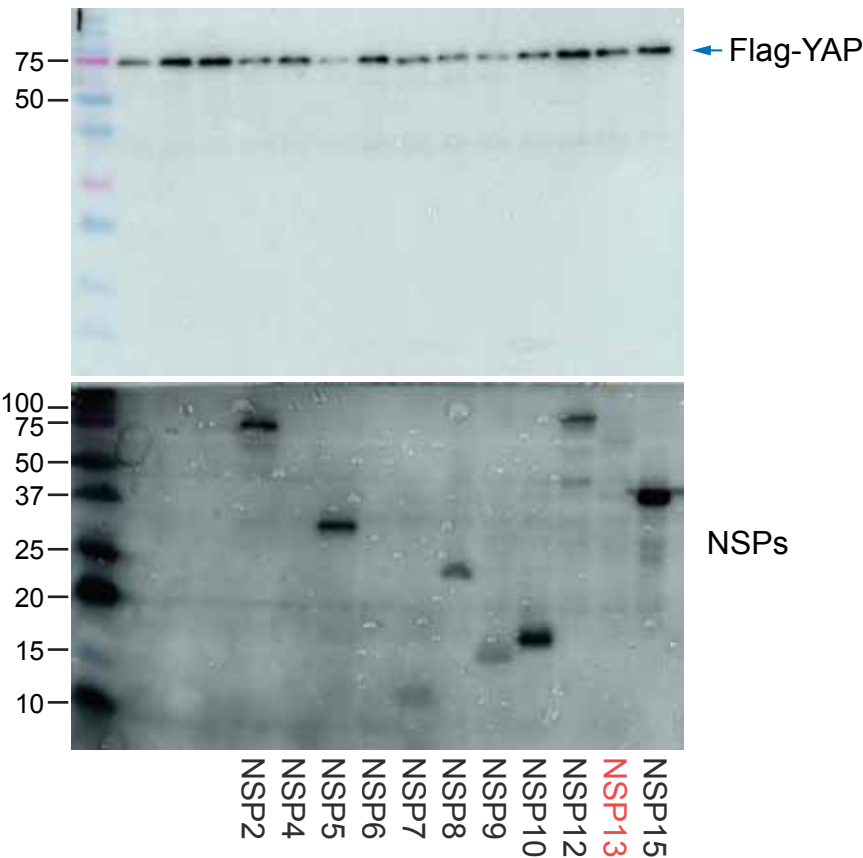

Supplement: Figure 2—source data 2. [file elife-100248-fig2-data2.zip › Figure 2A.pdf]

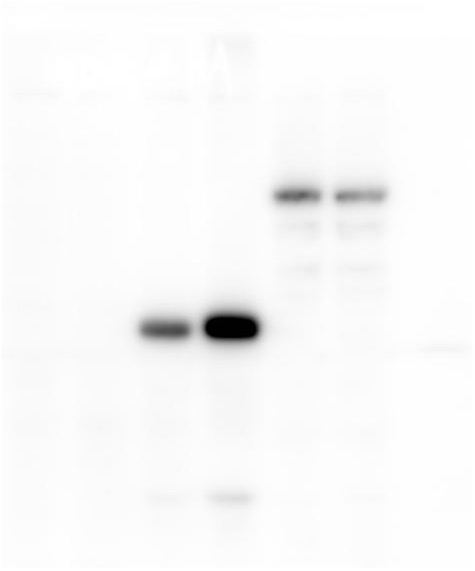

Supplement: Figure 2—source data 3. [file elife-100248-fig2-data3.zip › HA-NSP13 and Lats2 WT, KR.jpg]

Figure 2B

150—  
100—  
75—

HA-Lats2/KR

HA-NSP13

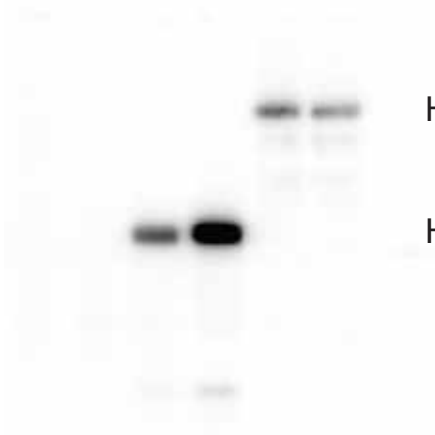

Supplement: Figure 2—source data 4. [file elife-100248-fig2-data4.zip › Figure 2B.pdf]

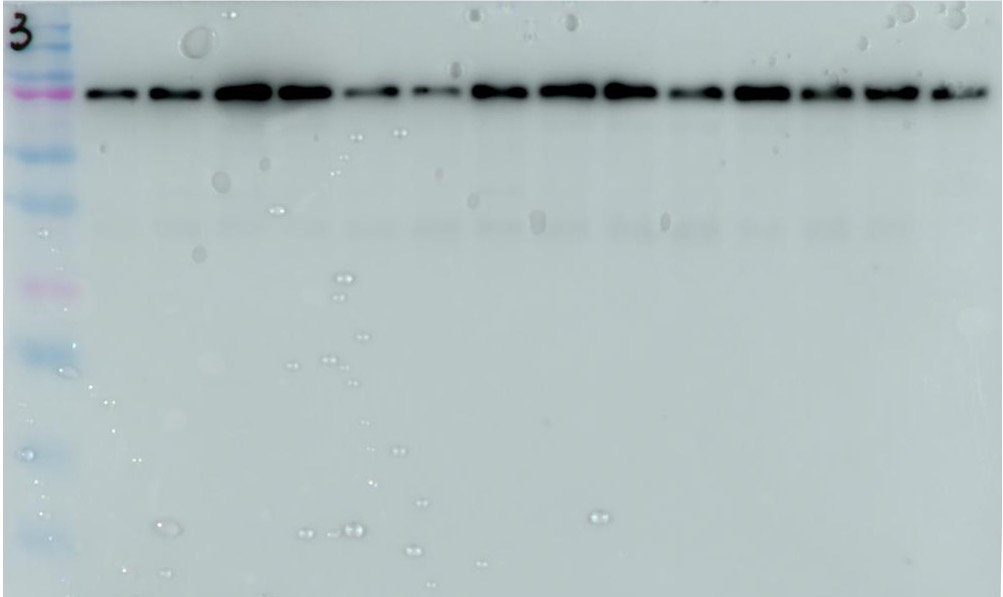

Supplement: Figure 2—figure supplement 1—source data 1. [file elife-100248-fig2-figsupp1-data1.zip › Flag-YAP5SA.jpg]

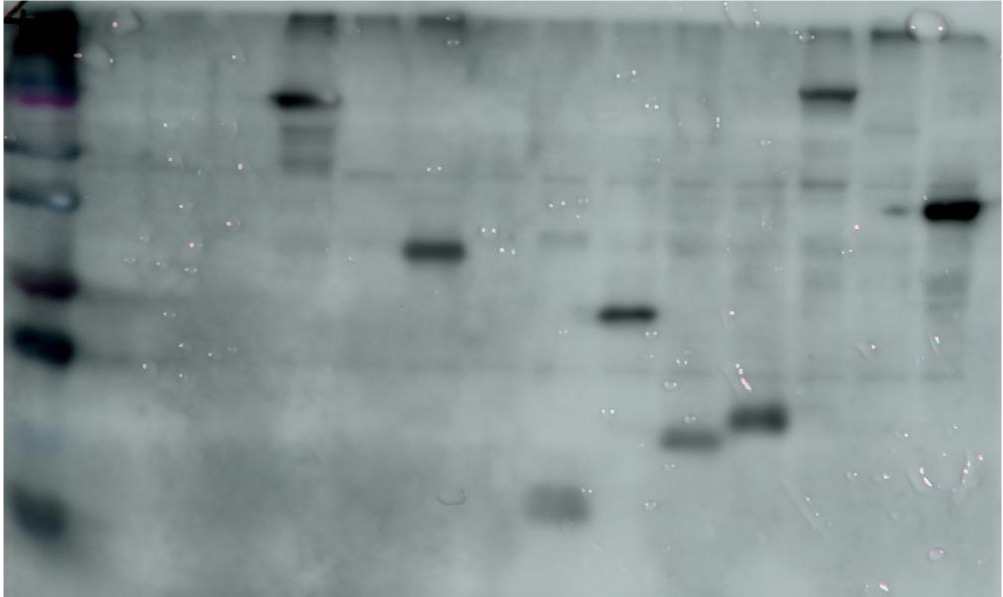

Supplement: Figure 2—figure supplement 1—source data 1. [file elife-100248-fig2-figsupp1-data1.zip › Strep-NSPs.jpg]

sFigure 2A

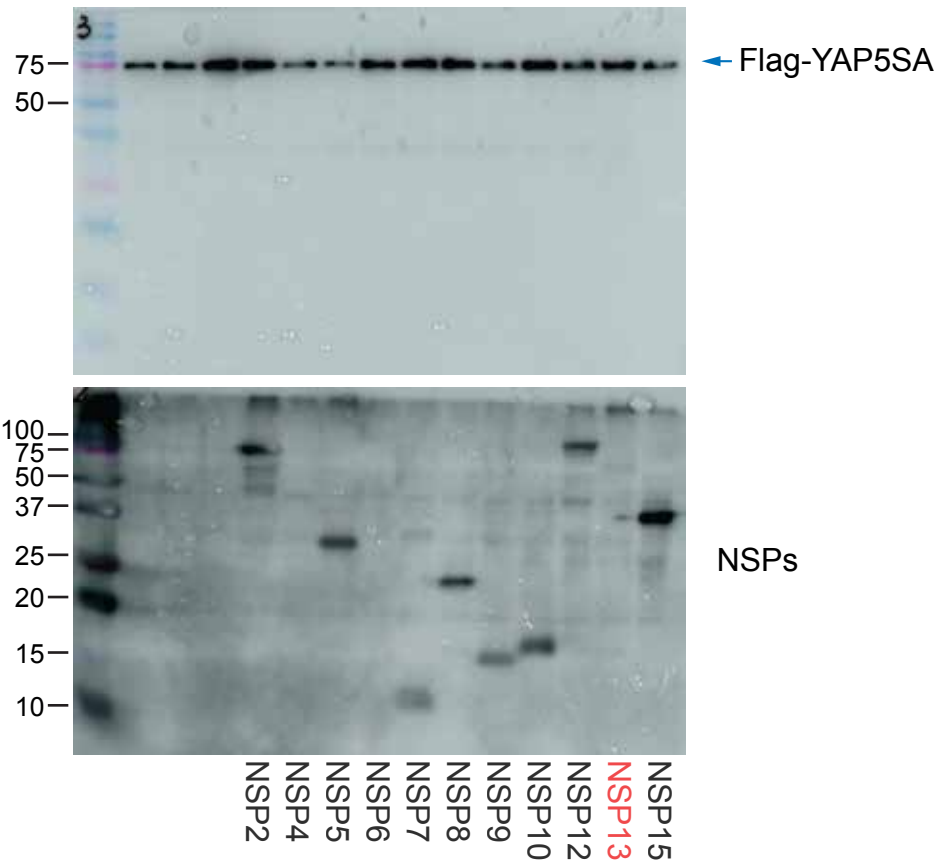

Supplement: Figure 2—figure supplement 1—source data 2. [file elife-100248-fig2-figsupp1-data2.zip › sFigure 2A.pdf]

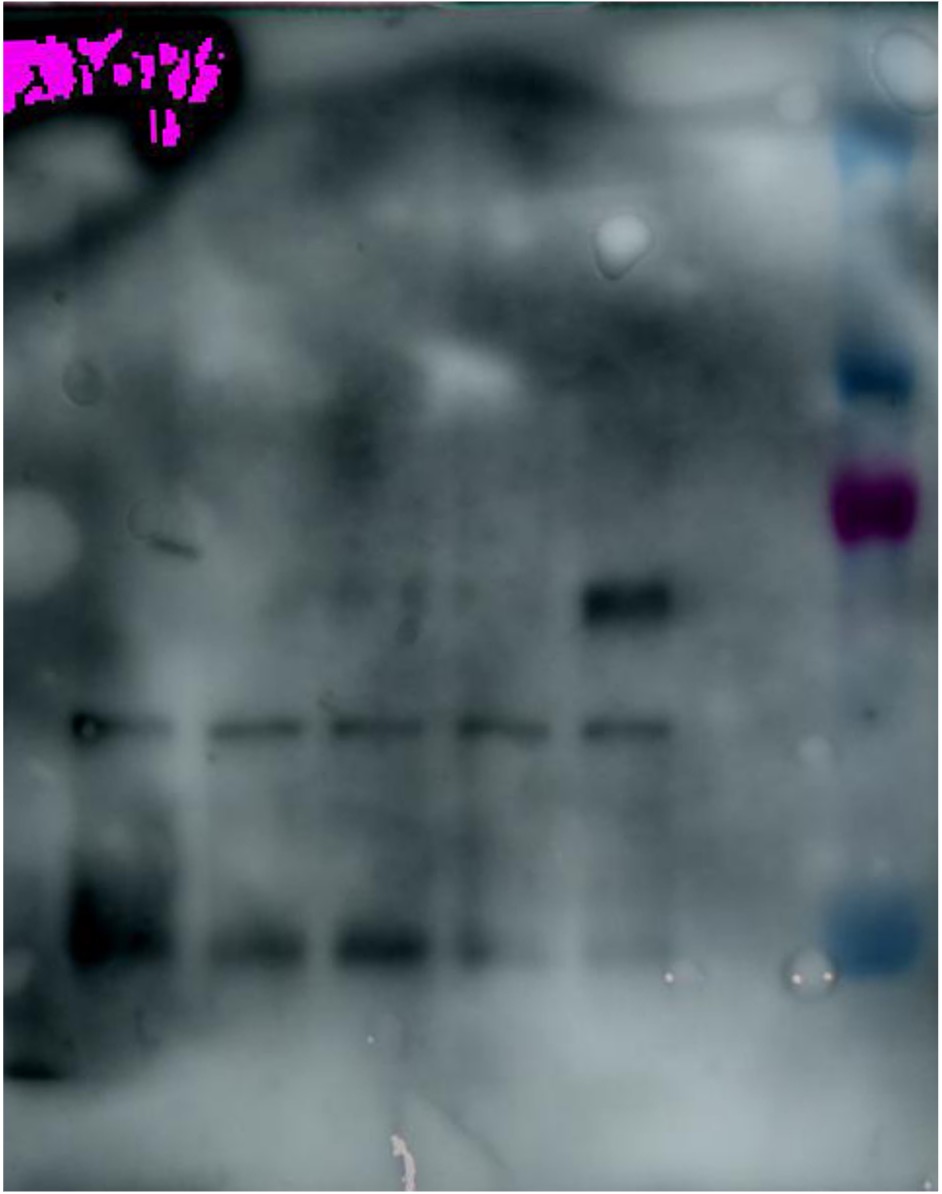

Supplement: Figure 2—figure supplement 4—source data 1. [file elife-100248-fig2-figsupp4-data1.zip › HA-NSP13.jpg]

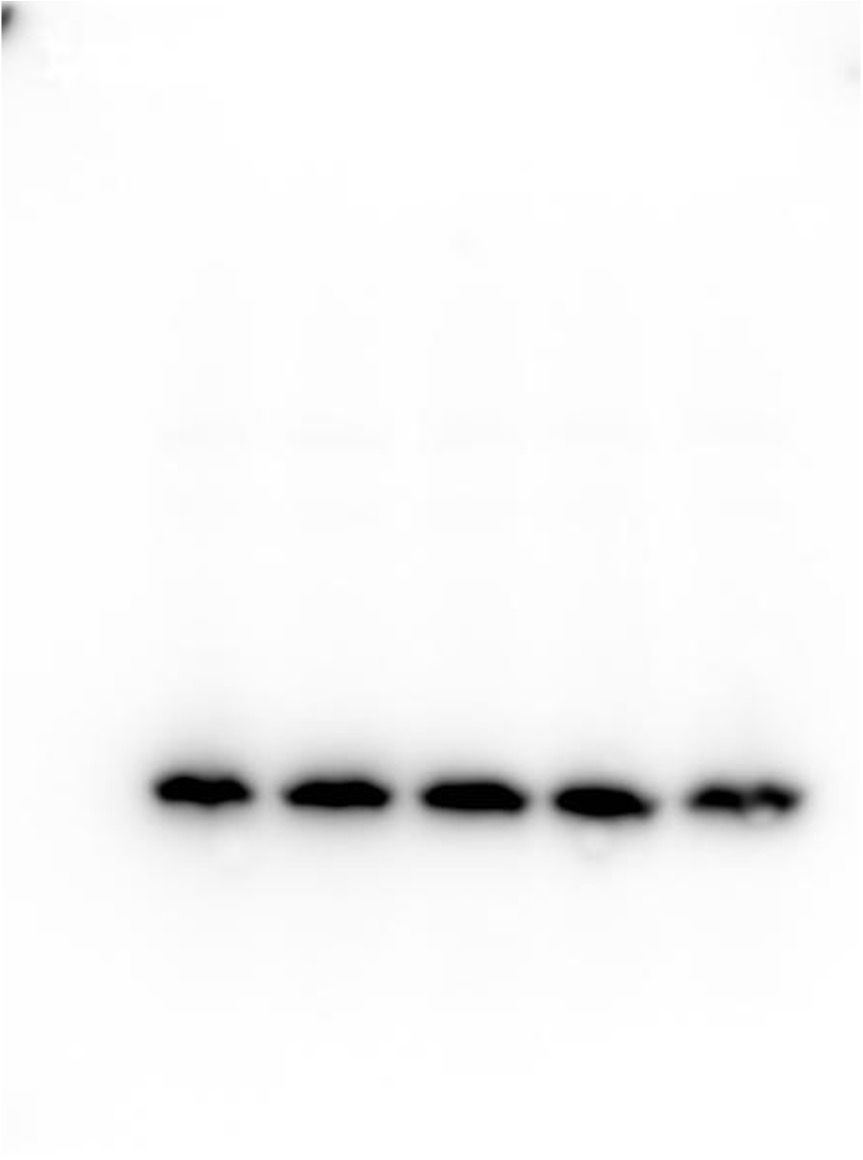

Supplement: Figure 2—figure supplement 4—source data 1. [file elife-100248-fig2-figsupp4-data1.zip › b-actin.jpg]

# sFigure 2D

| Days PVI   | 12 | 3 | 6 | 9 | 12 |
|------------|----|---|---|---|----|
| AAV9-GFP   | +  | - | - | - | -  |
| AAV9-NSP13 | -  | + | + | + | +  |

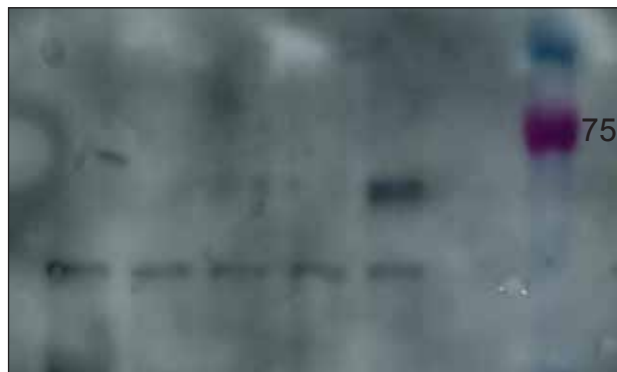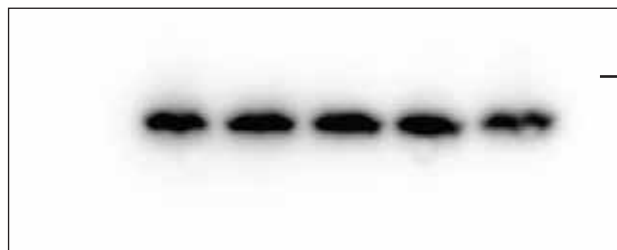

Heart lysis

Supplement: Figure 2—figure supplement 4—source data 2. [file elife-100248-fig2-figsupp4-data2.zip › sFigure 2D.pdf]

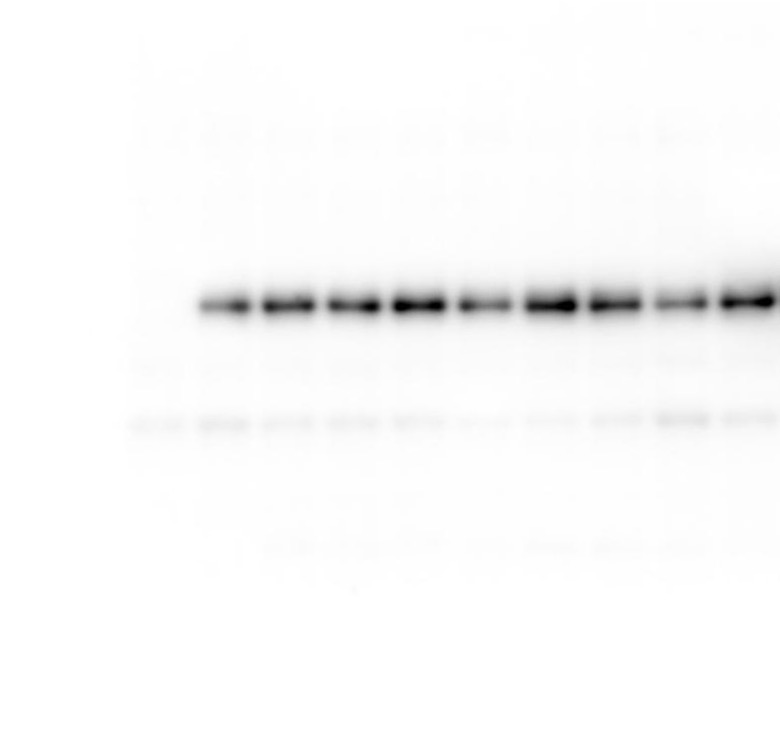

Supplement: Figure 3—source data 1. [file elife-100248-fig3-data1.zip › Flag-YAP5SA.jpg]

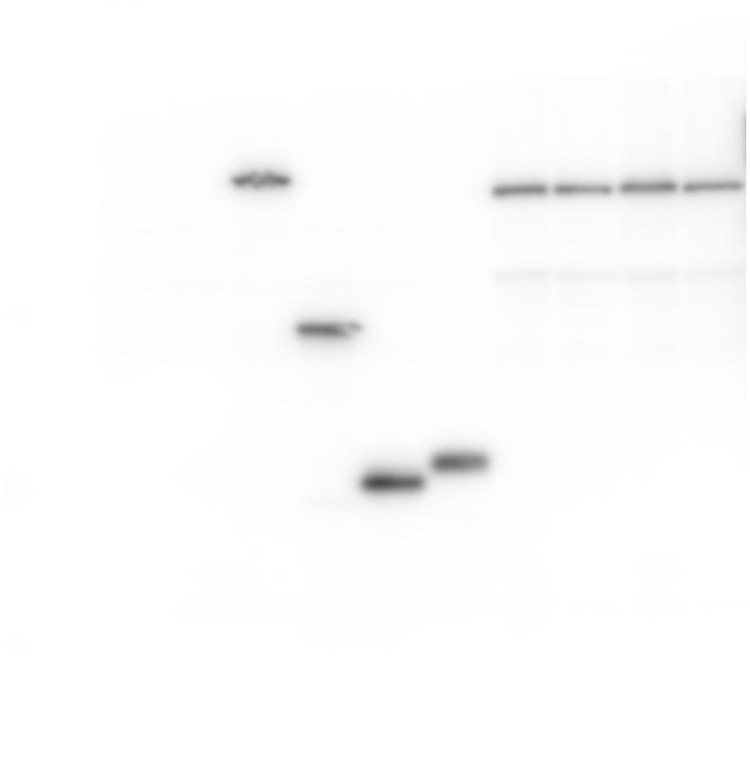

Supplement: Figure 3—source data 1. [file elife-100248-fig3-data1.zip › HA-NSPs.jpg]

### Figure 3B

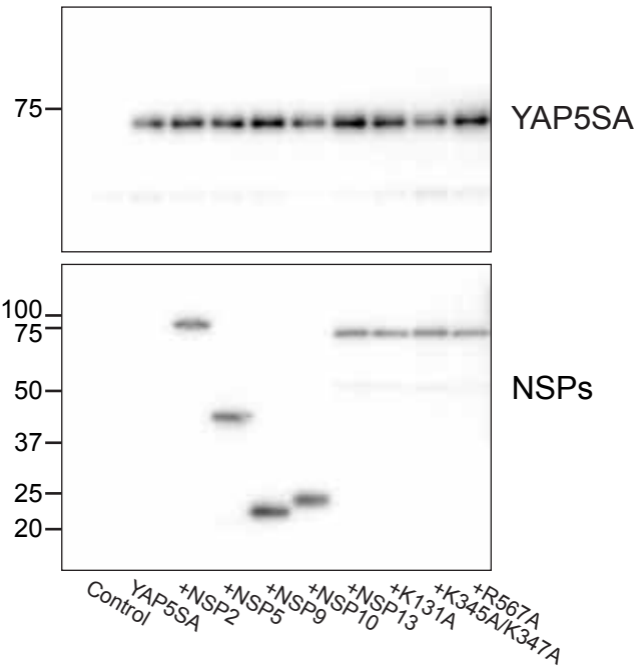

Supplement: Figure 3—source data 2. [file elife-100248-fig3-data2.zip › Figure 3B.pdf]

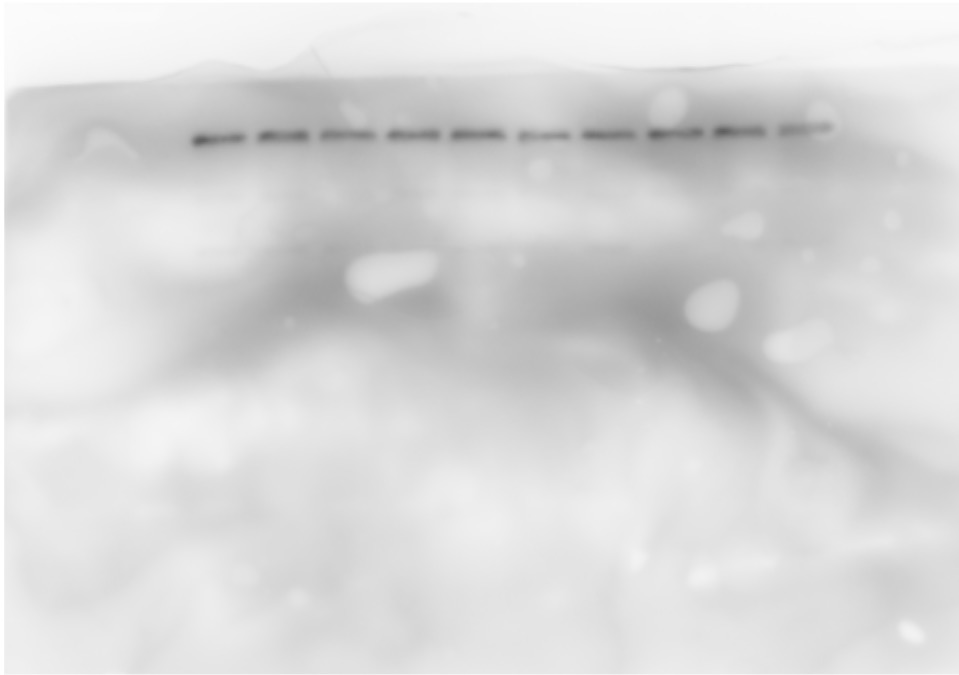

Supplement: Figure 3—source data 3. [file elife-100248-fig3-data3.zip › Flag-YAP5SA.jpg]

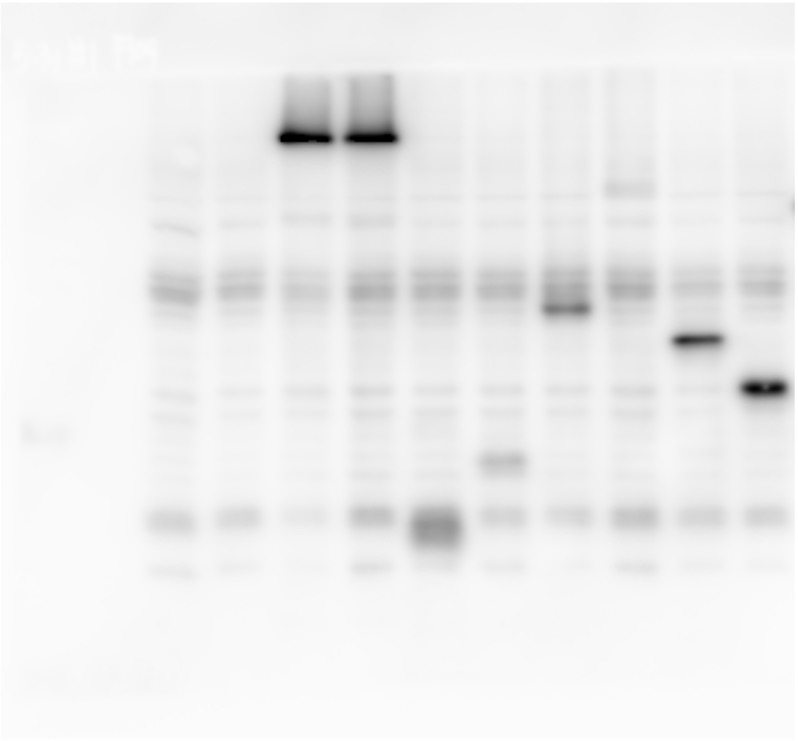

Supplement: Figure 3—source data 3. [file elife-100248-fig3-data3.zip › HA-NSP13 and truncations.jpg]

Figure 3D

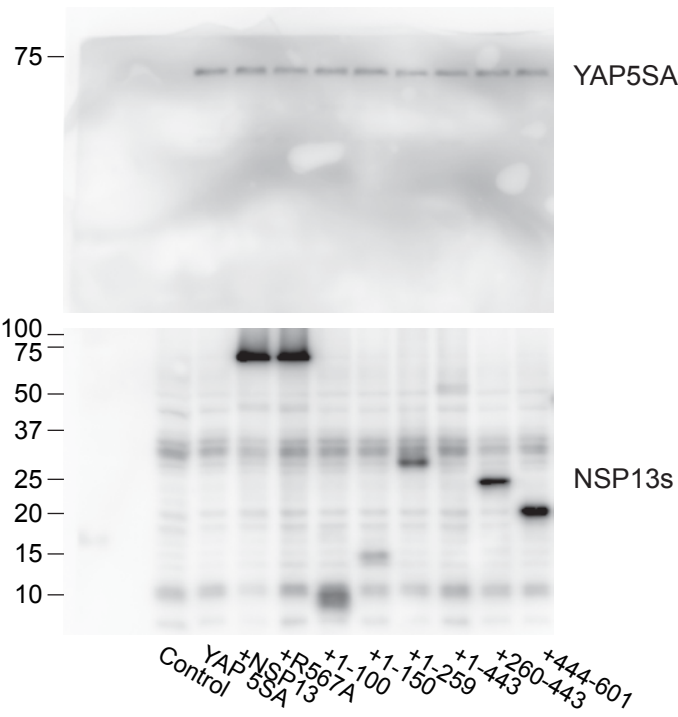

Supplement: Figure 3—source data 4. [file elife-100248-fig3-data4.zip › Figure 3D.pdf]

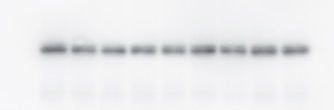

Supplement: Figure 3—source data 5. [file elife-100248-fig3-data5.zip › Flag-YAP5SA.jpg]

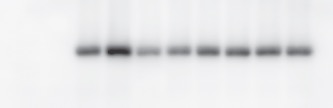

Supplement: Figure 3—source data 5. [file elife-100248-fig3-data5.zip › HA-NSP13s.jpg]

Figure 3F

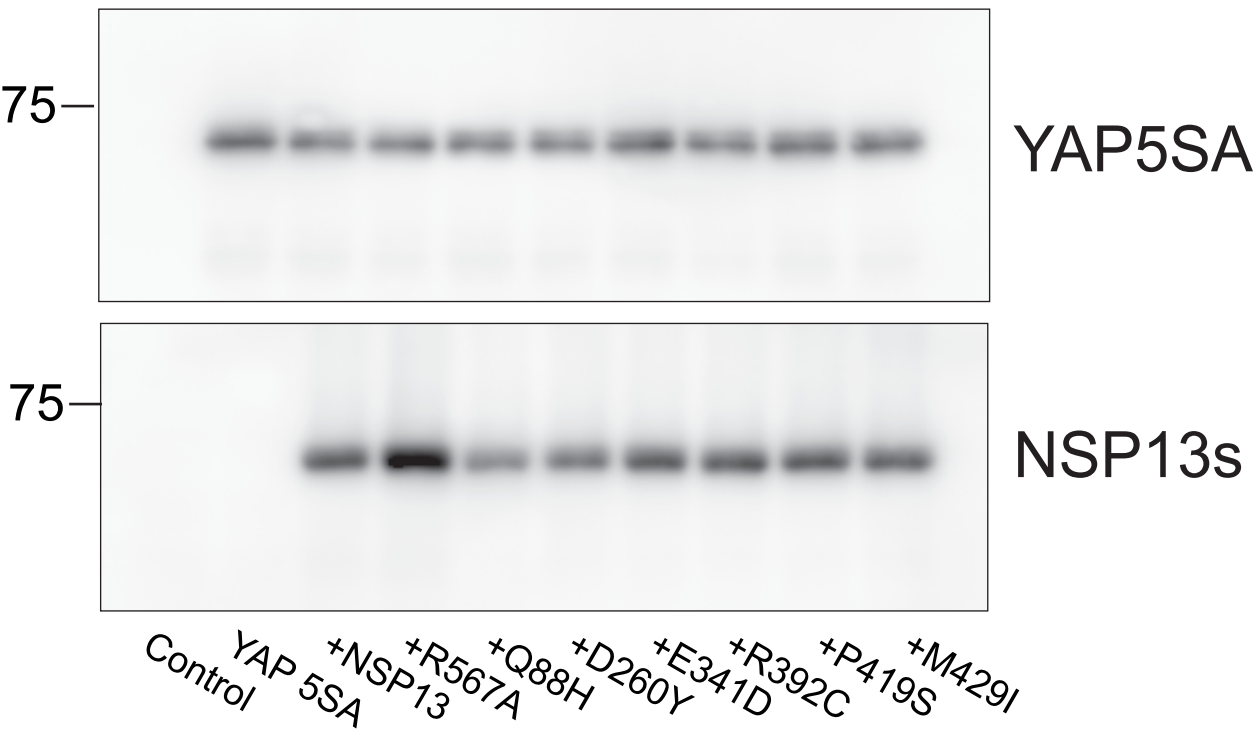

Supplement: Figure 3—source data 6. [file elife-100248-fig3-data6.zip › Figure 3F.pdf]

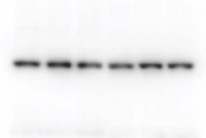

Supplement: Figure 4—source data 1. [file elife-100248-fig4-data1.zip › Myc-TEAD4 in WCL.jpg]

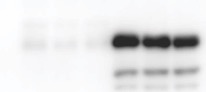

Supplement: Figure 4—source data 1. [file elife-100248-fig4-data1.zip › GAPDH in WCL.jpg]

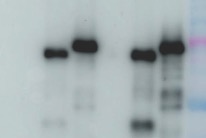

Supplement: Figure 4—source data 1. [file elife-100248-fig4-data1.zip › HA-NSP2 and NSP13 in IP.jpg]

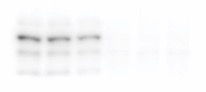

Supplement: Figure 4—source data 1. [file elife-100248-fig4-data1.zip › Lamin AC in WCL.jpg]

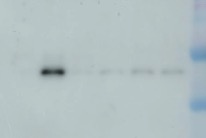

Supplement: Figure 4—source data 1. [file elife-100248-fig4-data1.zip › Myc-TEAD4 in IP.jpg]

Figure 4B

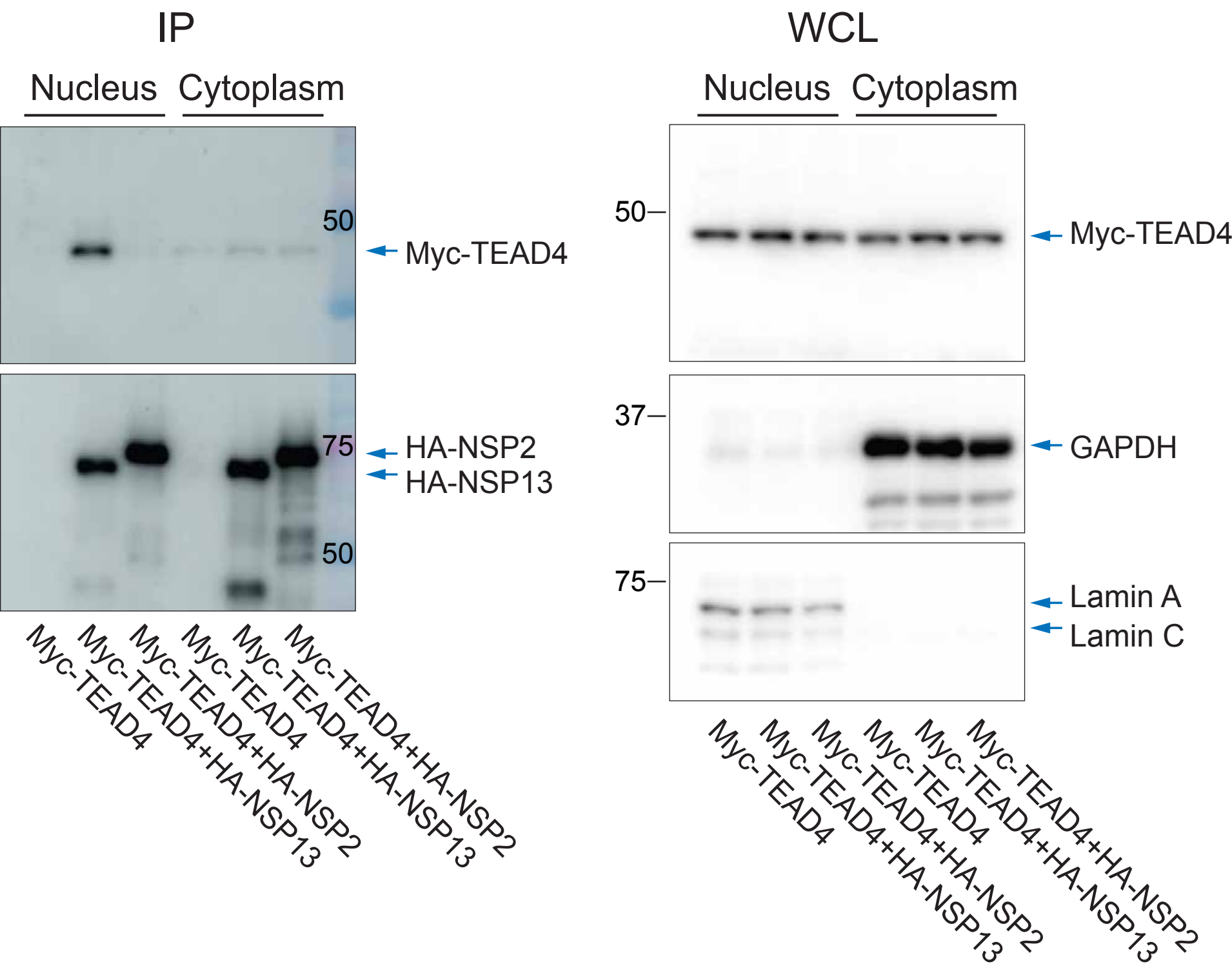

Supplement: Figure 4—source data 2. [file elife-100248-fig4-data2.zip › Figure 4B.pdf]

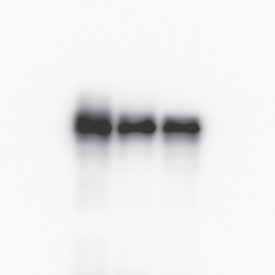

Supplement: Figure 4—source data 3. [file elife-100248-fig4-data3.zip › Flag-YAP WT in IP.jpg]

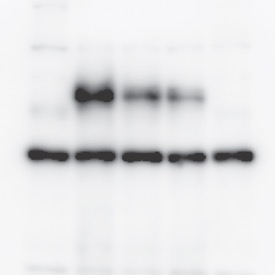

Supplement: Figure 4—source data 3. [file elife-100248-fig4-data3.zip › Flag-YAP WT in WCL.jpg]

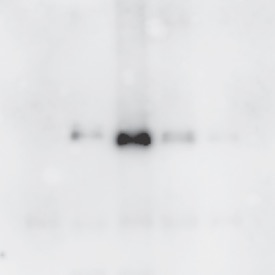

Supplement: Figure 4—source data 3. [file elife-100248-fig4-data3.zip › HA-NSP13 in IP.jpg]

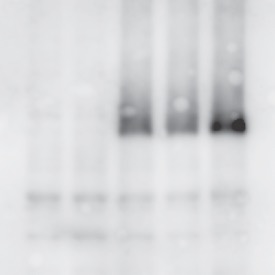

Supplement: Figure 4—source data 3. [file elife-100248-fig4-data3.zip › HA-NSP13 in WCL.jpg]

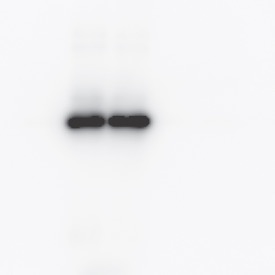

Supplement: Figure 4—source data 3. [file elife-100248-fig4-data3.zip › Myc-TEAD4 in IP.jpg]

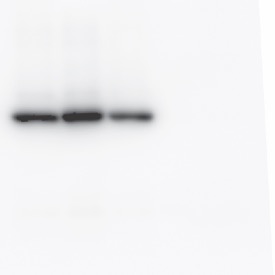

Supplement: Figure 4—source data 3. [file elife-100248-fig4-data3.zip › Myc-TEAD4 in WCL.jpg]

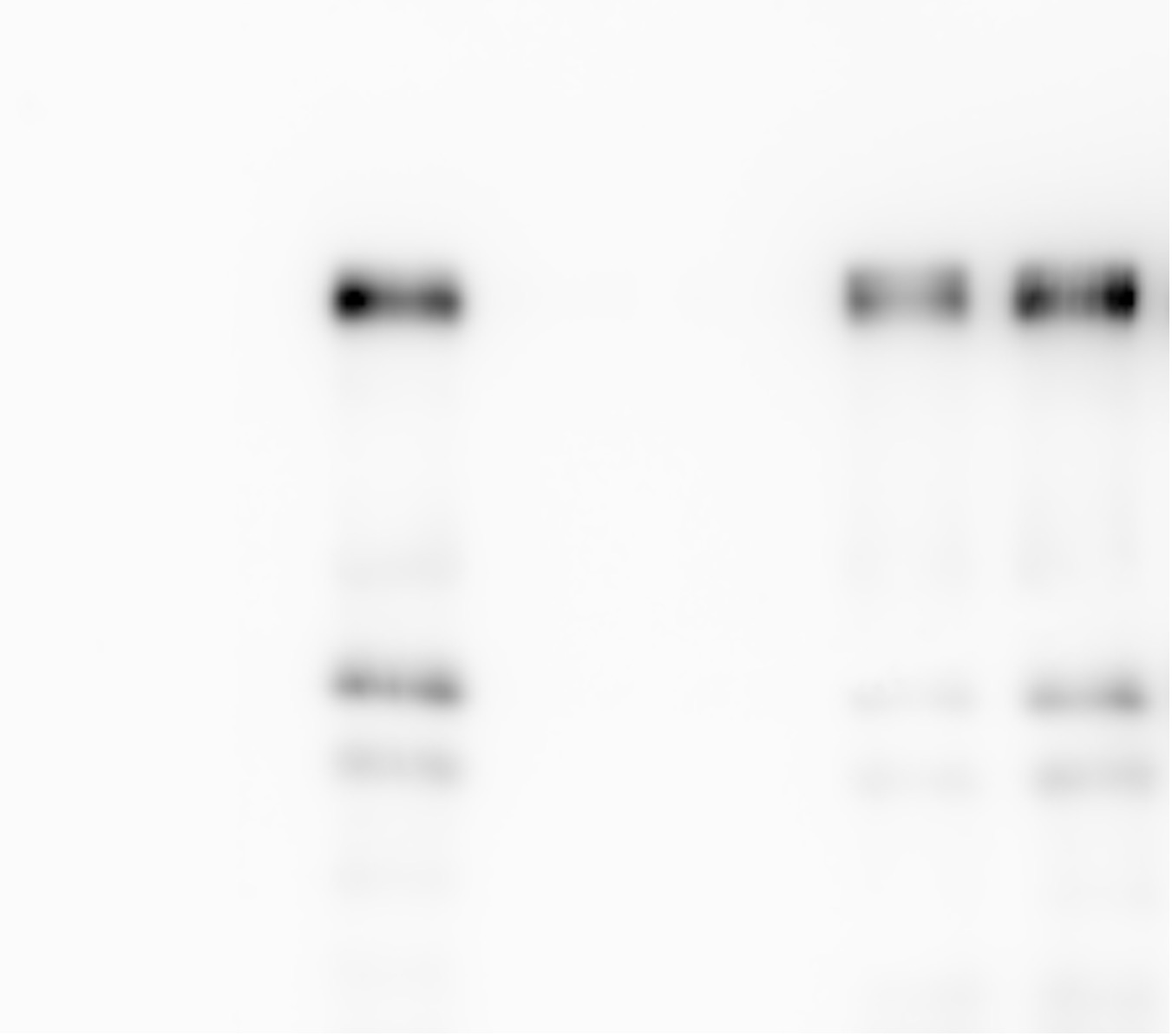

Supplement: Figure 4—source data 5. [file elife-100248-fig4-data5.zip › Flag-YAP5SA in IP.jpg]

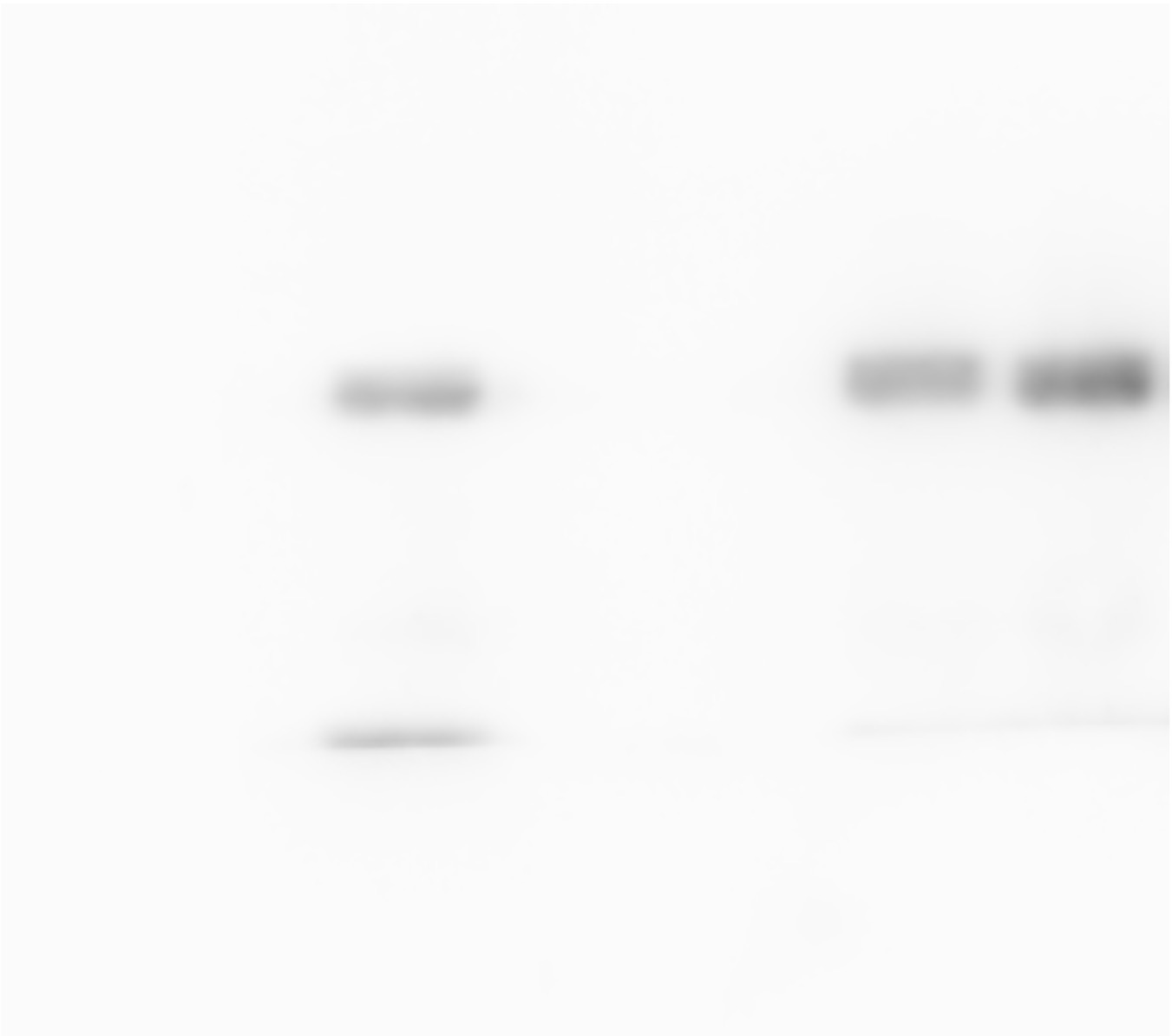

Supplement: Figure 4—source data 5. [file elife-100248-fig4-data5.zip › Flag-YAP5SA in WCL.jpg]

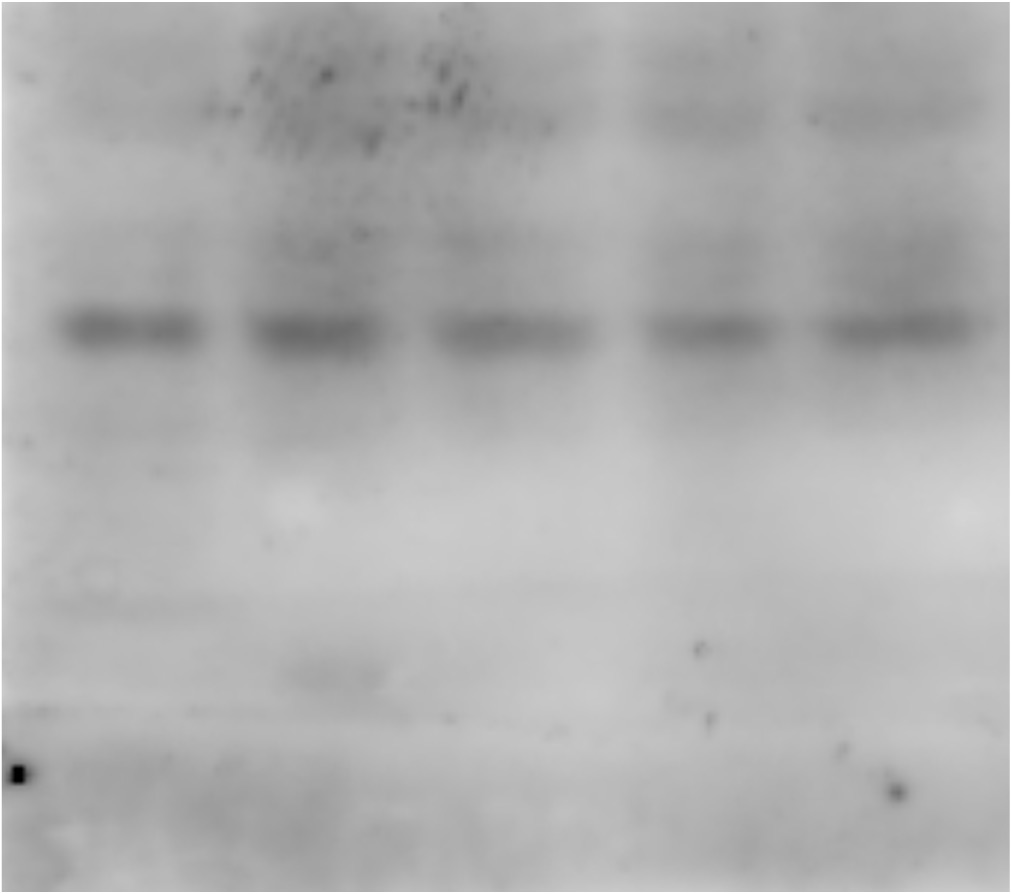

Supplement: Figure 4—source data 5. [file elife-100248-fig4-data5.zip › GAPDH in WCL.jpg]

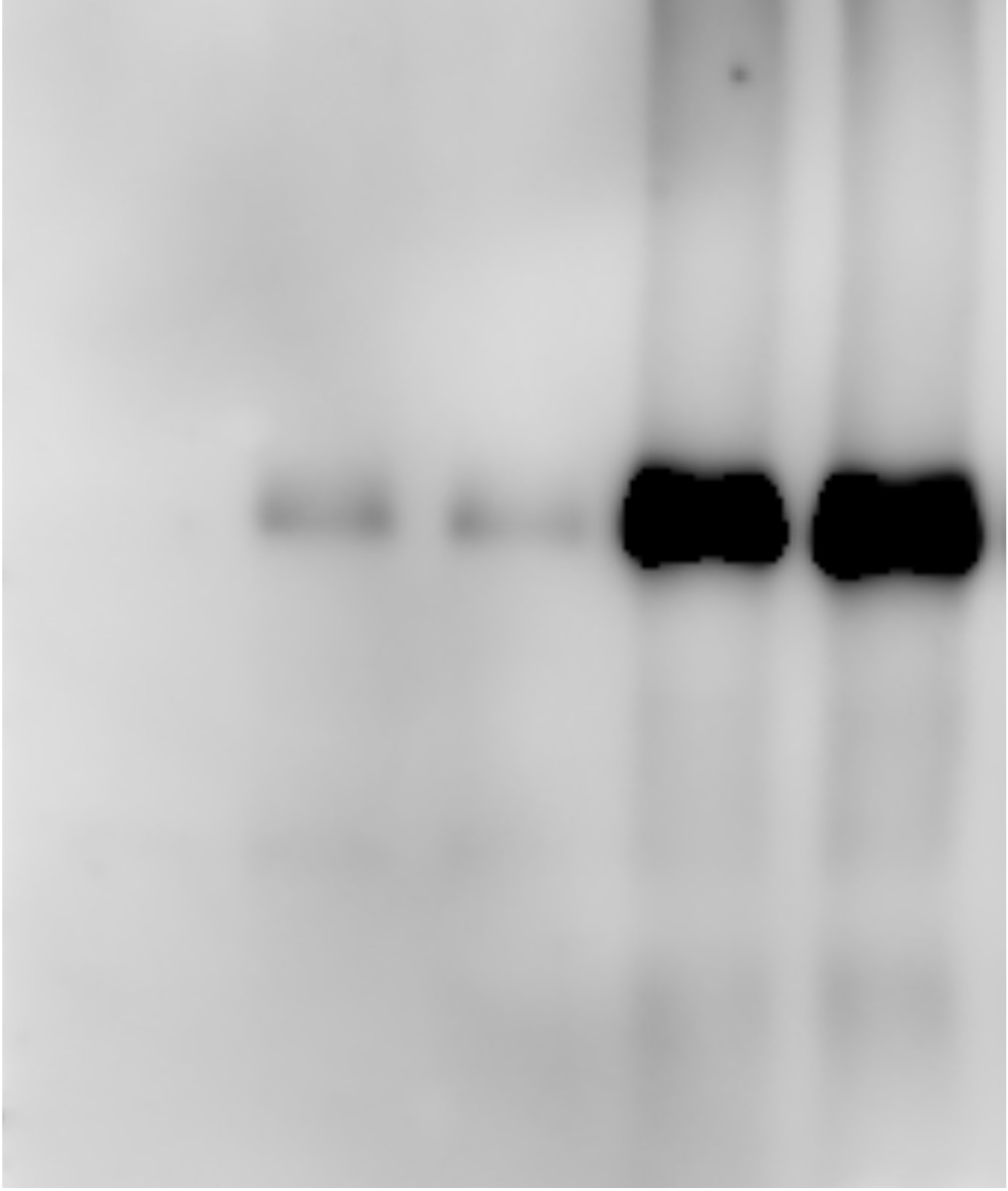

Supplement: Figure 4—source data 5. [file elife-100248-fig4-data5.zip › HA-NSP13 in IP long exposure.jpg]

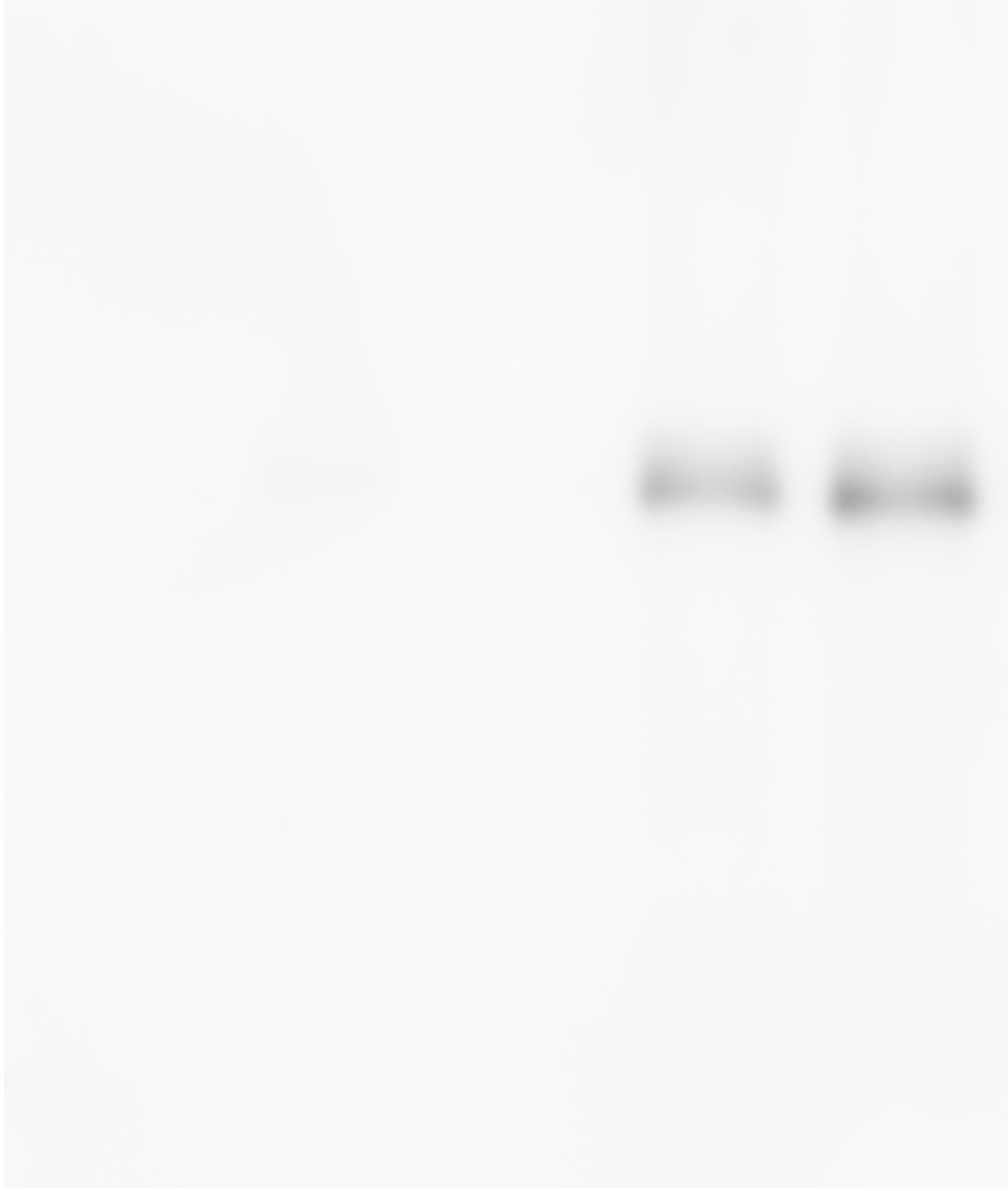

Supplement: Figure 4—source data 5. [file elife-100248-fig4-data5.zip › HA-NSP13 in IP.jpg]

Figure 4E

|             |   |   |   |   |   |
|-------------|---|---|---|---|---|
| NSP13 virus | - | + | + | + | + |
| TAM (Days)  | 3 | - | - | 3 | 3 |

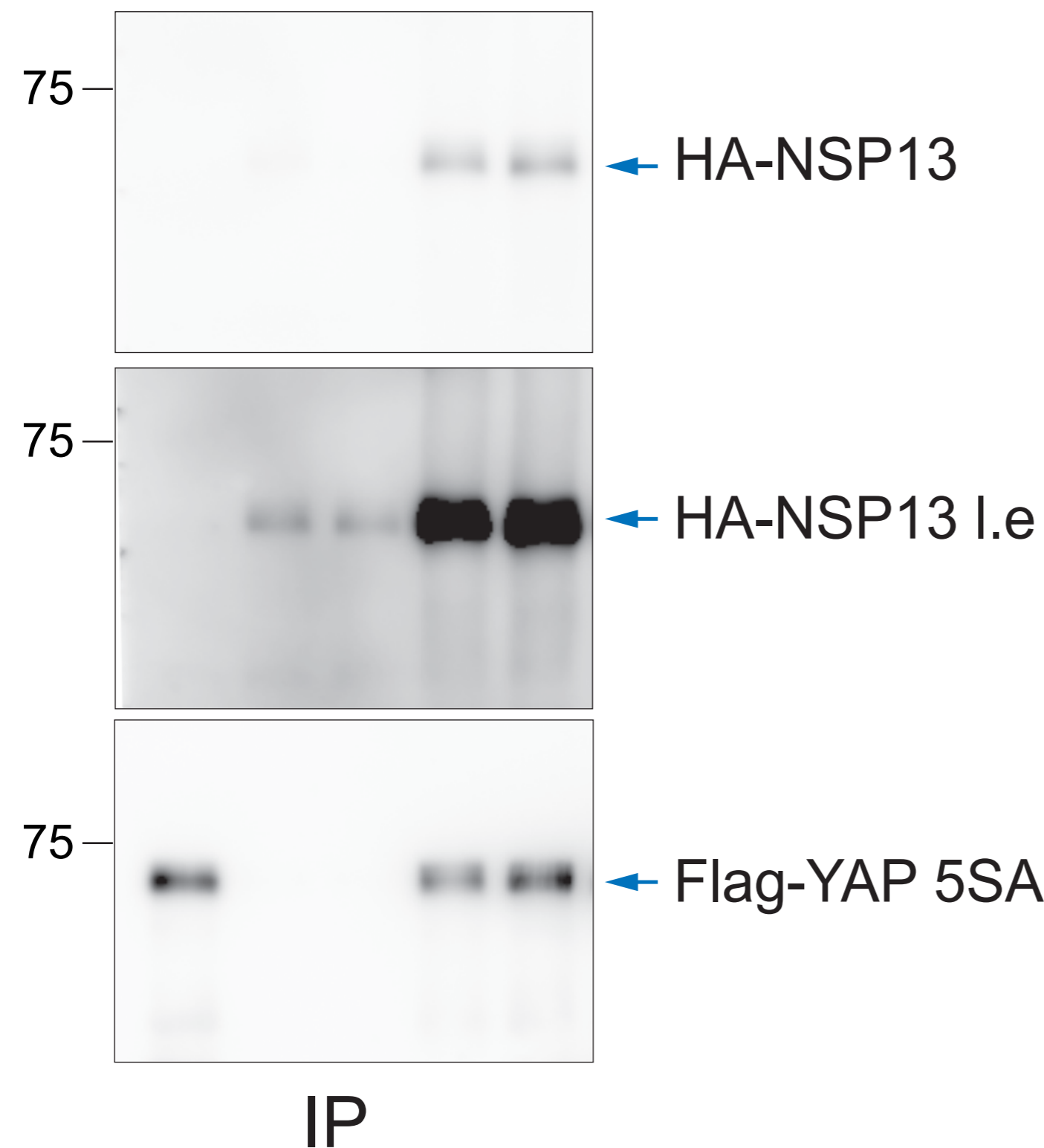

|             |   |   |   |   |   |
|-------------|---|---|---|---|---|
| NSP13 virus | - | + | + | + | + |
| TAM (Days)  | 3 | - | - | 3 | 3 |

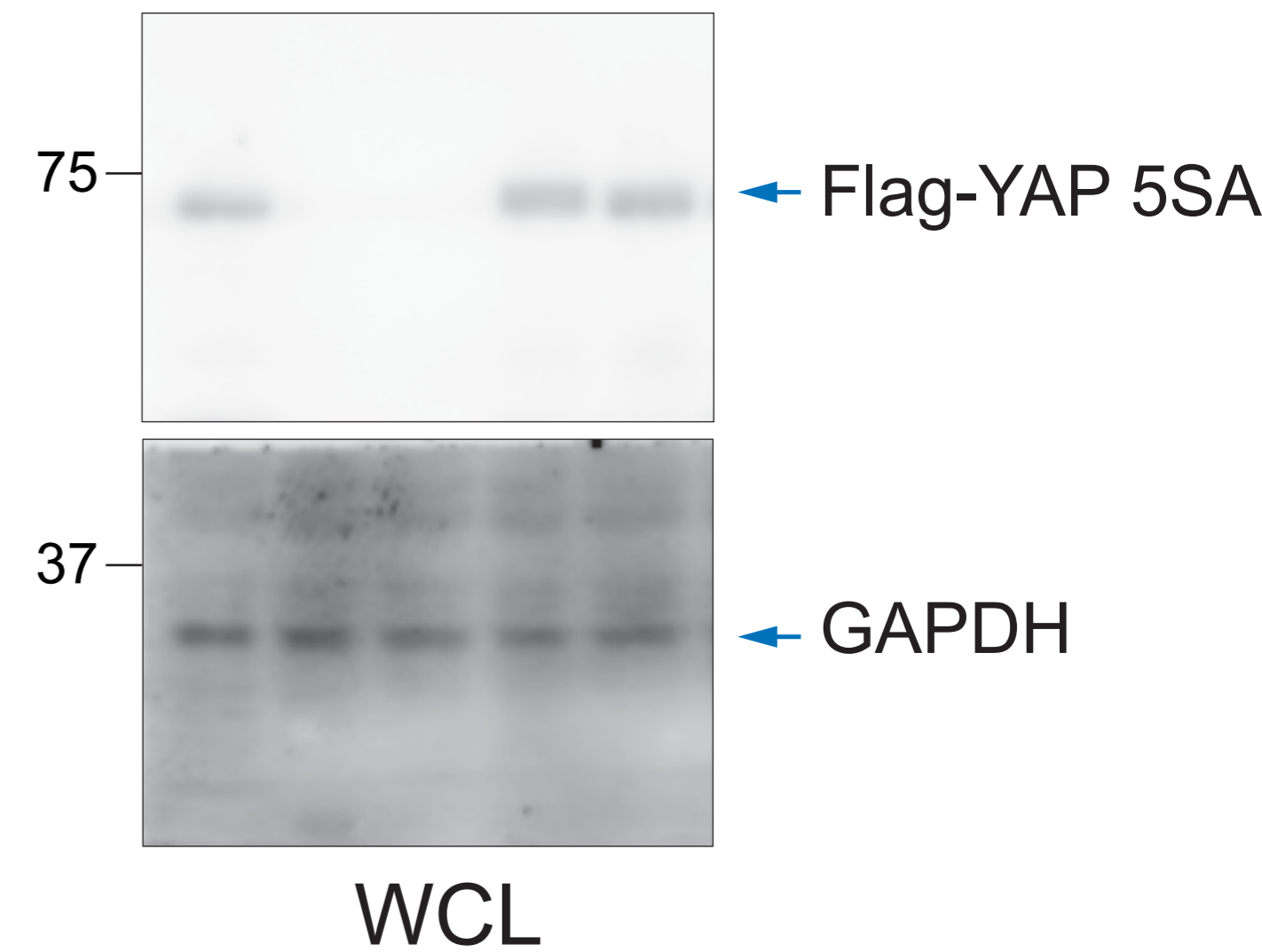

Supplement: Figure 4—source data 6. [file elife-100248-fig4-data6.zip › Figure 4E.pdf]

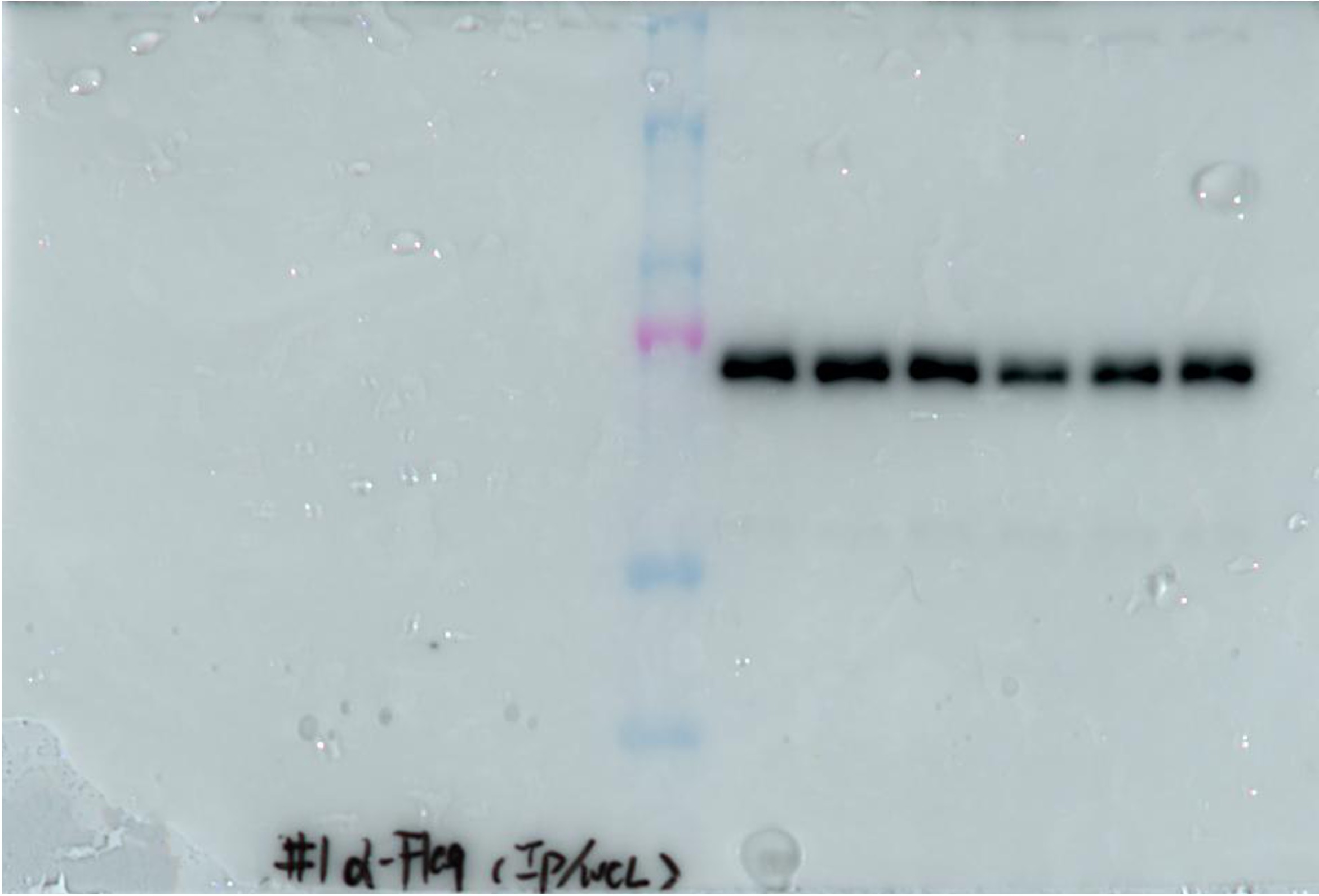

Supplement: Figure 4—figure supplement 1—source data 1. [file elife-100248-fig4-figsupp1-data1.zip › Flag-YAP5SA in IP and WCL.jpg]

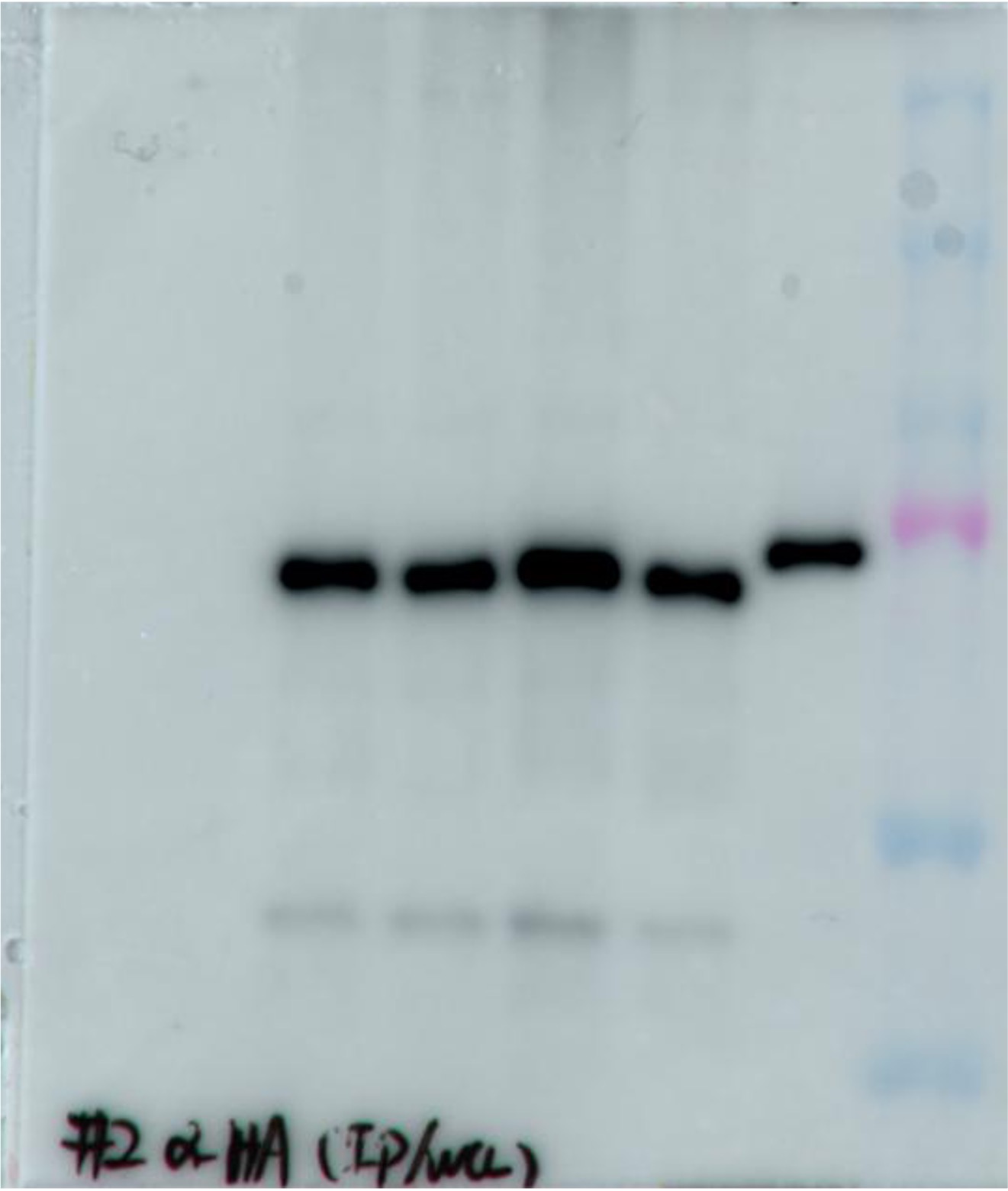

Supplement: Figure 4—figure supplement 1—source data 1. [file elife-100248-fig4-figsupp1-data1.zip › HA-NSP13s in IP.jpg]

sFigure 4A

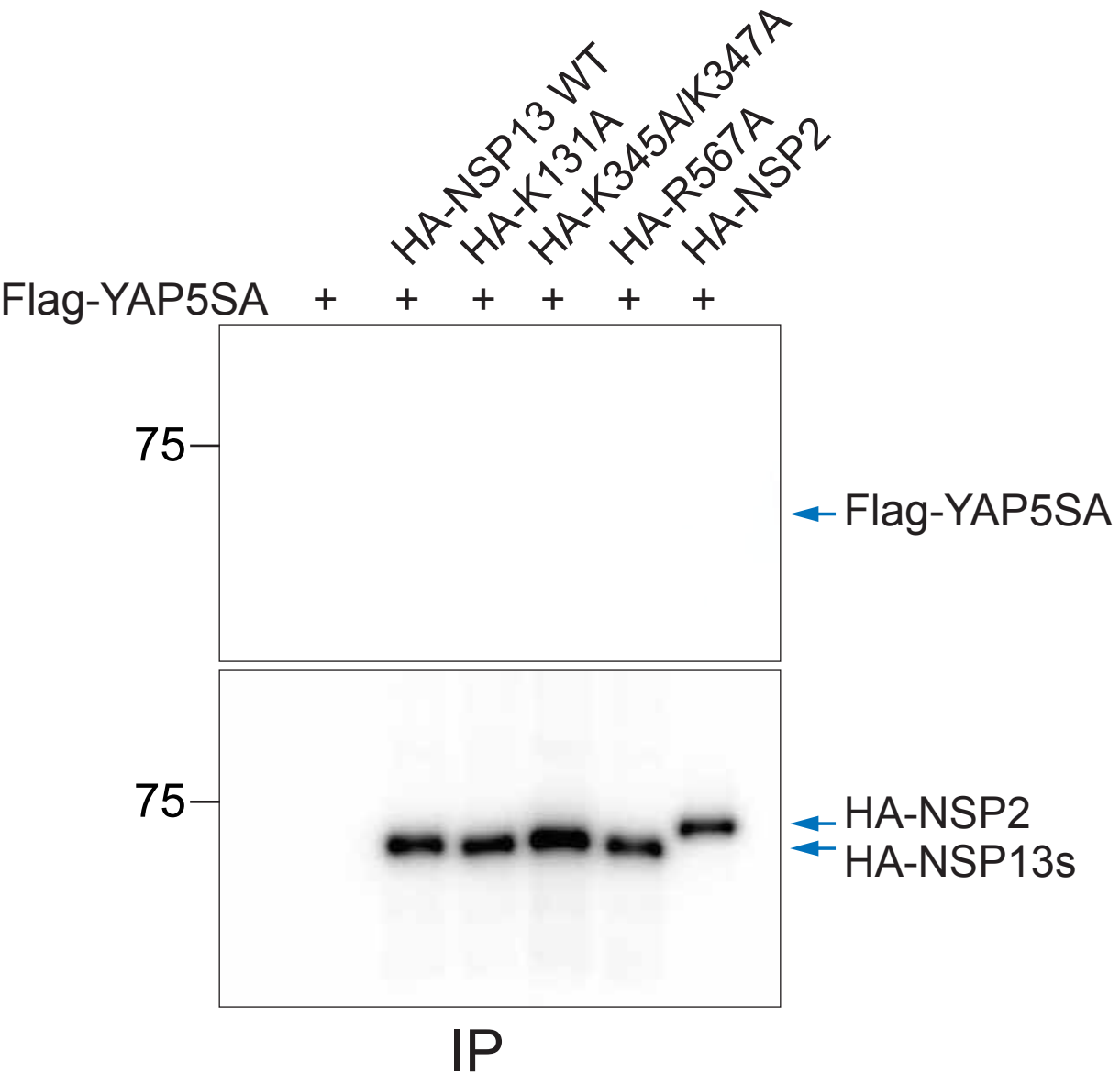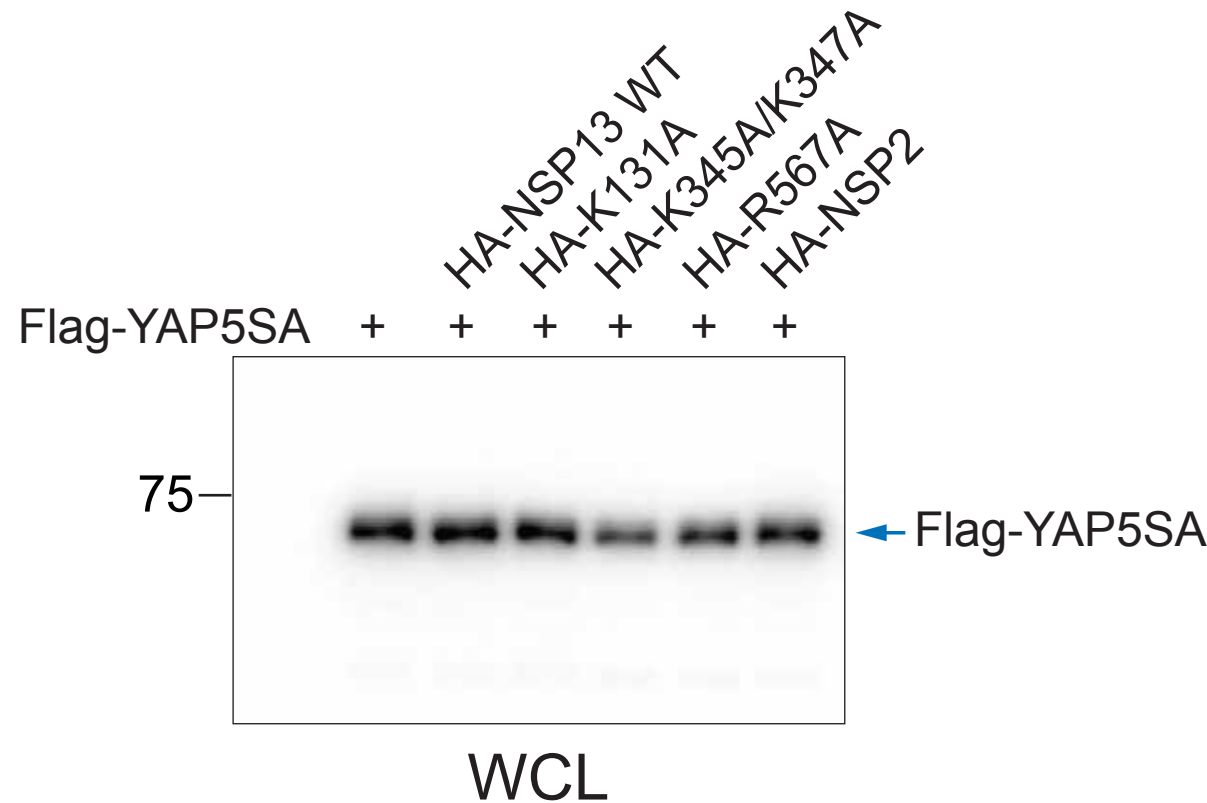

Supplement: Figure 4—figure supplement 1—source data 2. [file elife-100248-fig4-figsupp1-data2.zip › sFigure 4A.pdf]

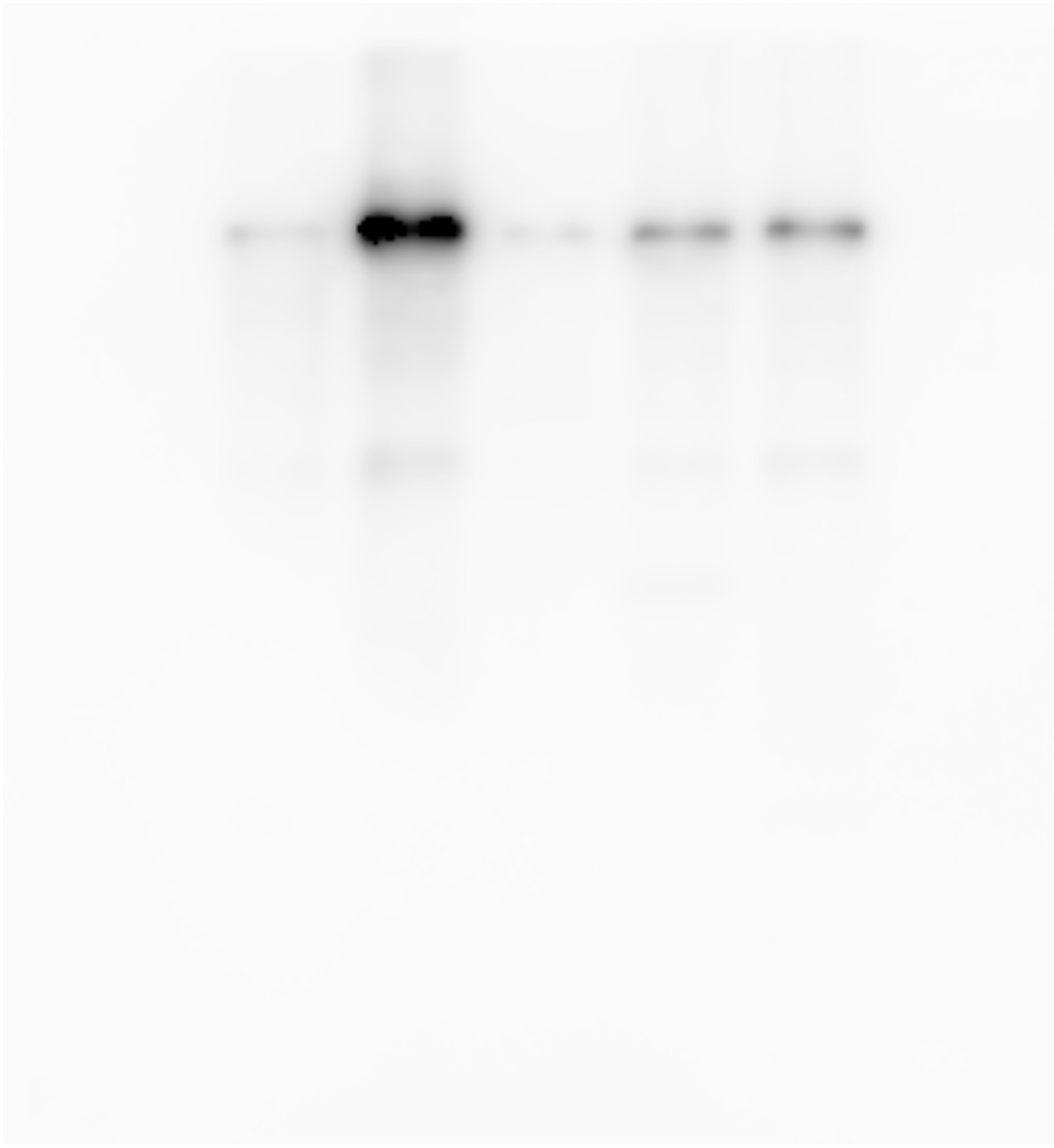

Supplement: Figure 4—figure supplement 2—source data 1. [file elife-100248-fig4-figsupp2-data1.zip › HA-NSP13 in IP.jpg]

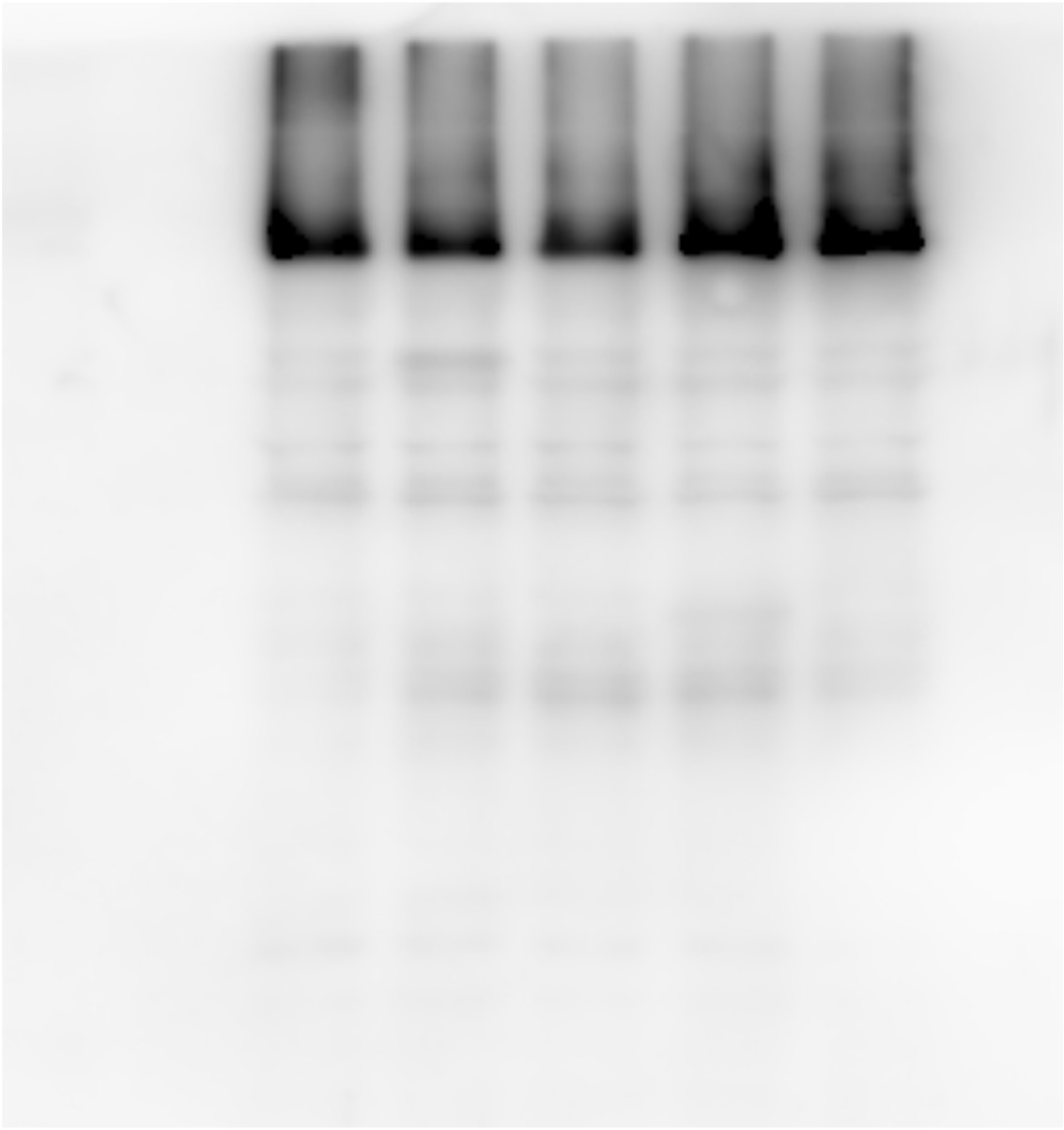

Supplement: Figure 4—figure supplement 2—source data 1. [file elife-100248-fig4-figsupp2-data1.zip › HA-NSP13 in WCL.jpg]

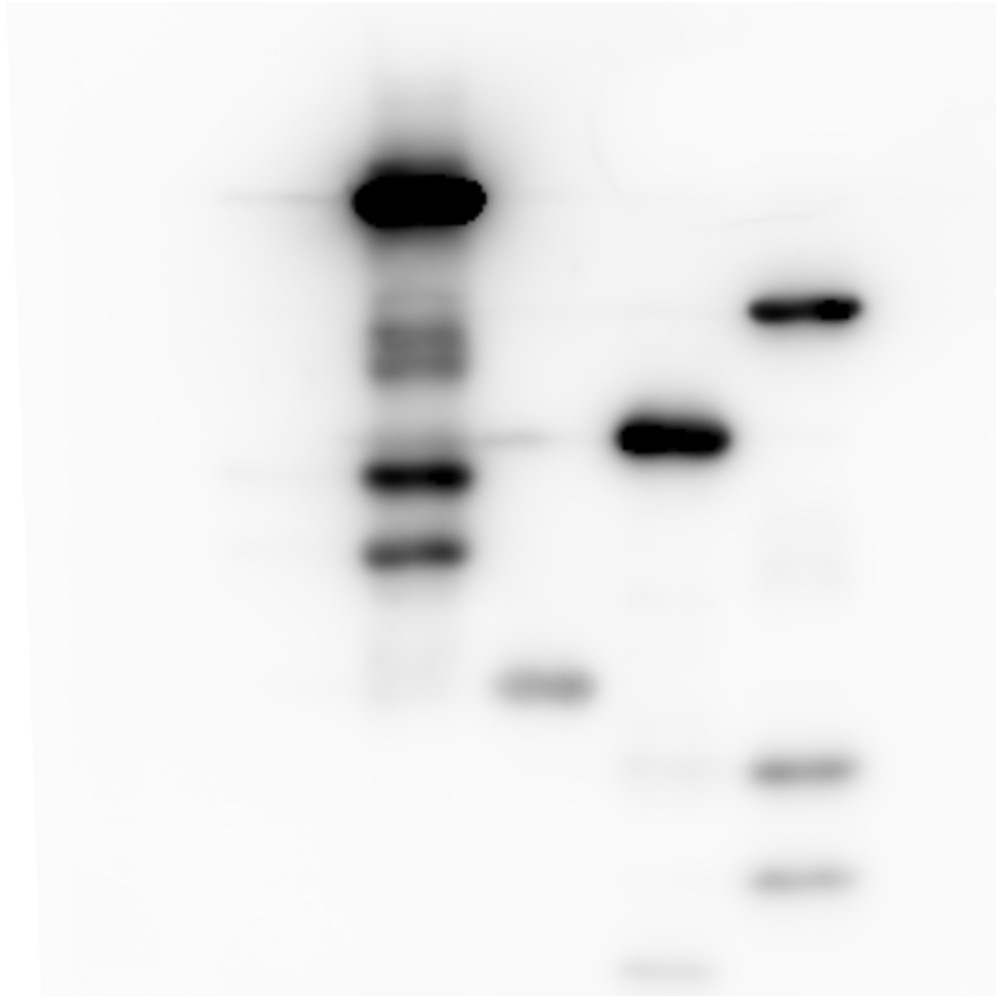

Supplement: Figure 4—figure supplement 2—source data 1. [file elife-100248-fig4-figsupp2-data1.zip › Myc-TEAD4 and truncations in IP.jpg]

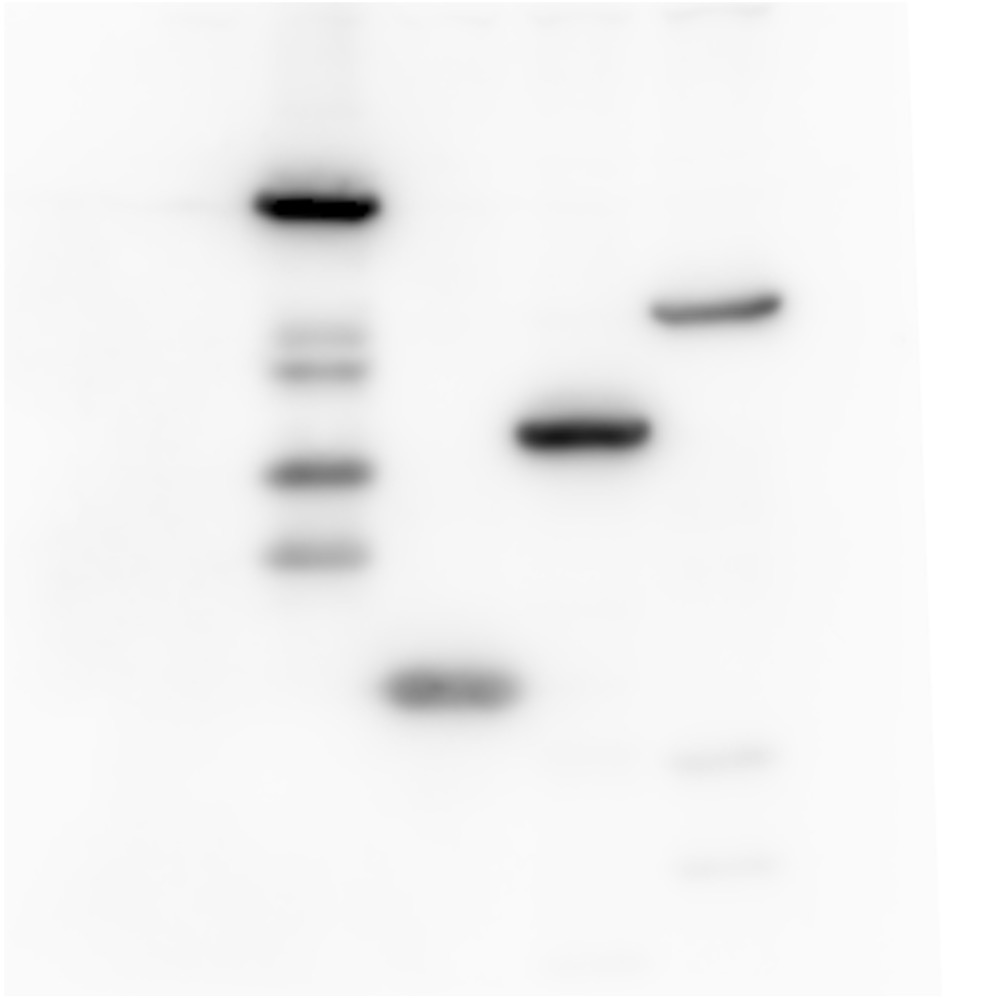

Supplement: Figure 4—figure supplement 2—source data 1. [file elife-100248-fig4-figsupp2-data1.zip › Myc-TEAD4 and truncations in WCL.jpg]

sFigure 4B

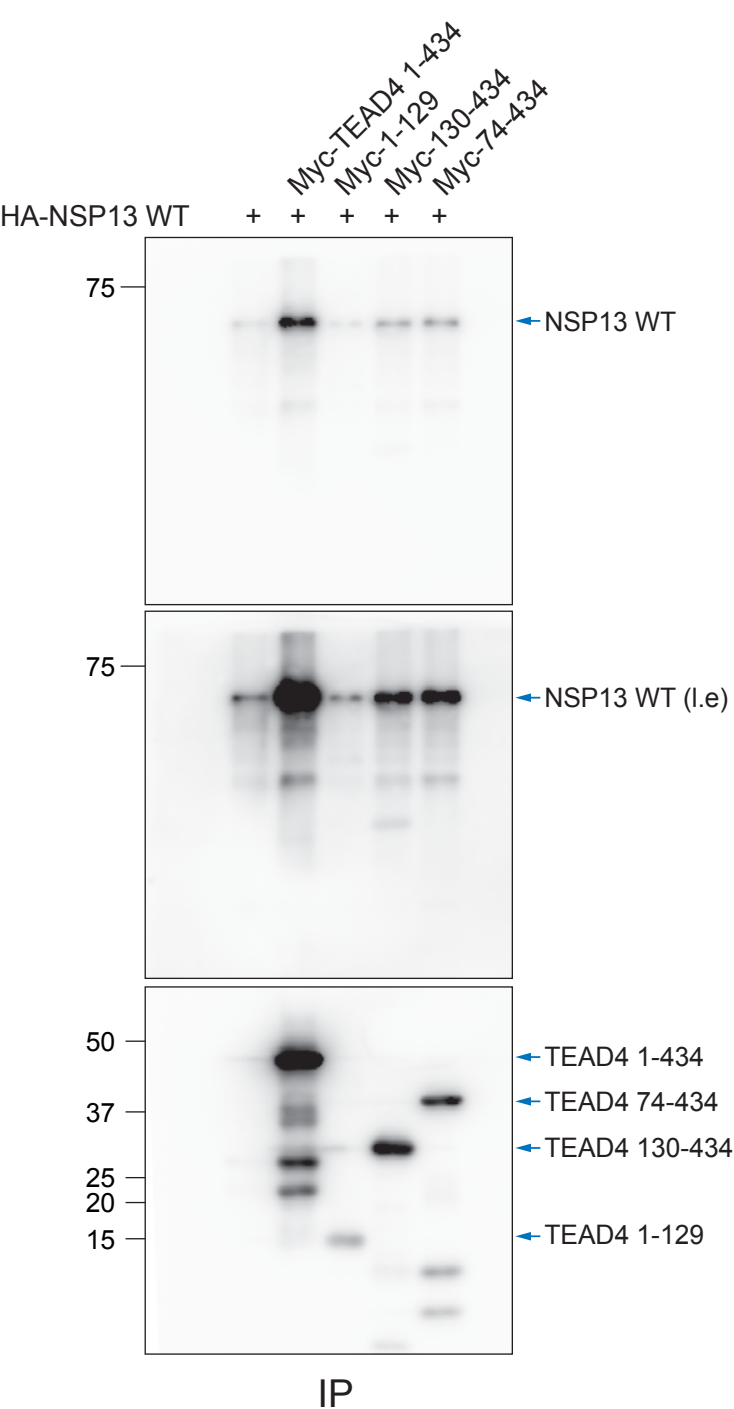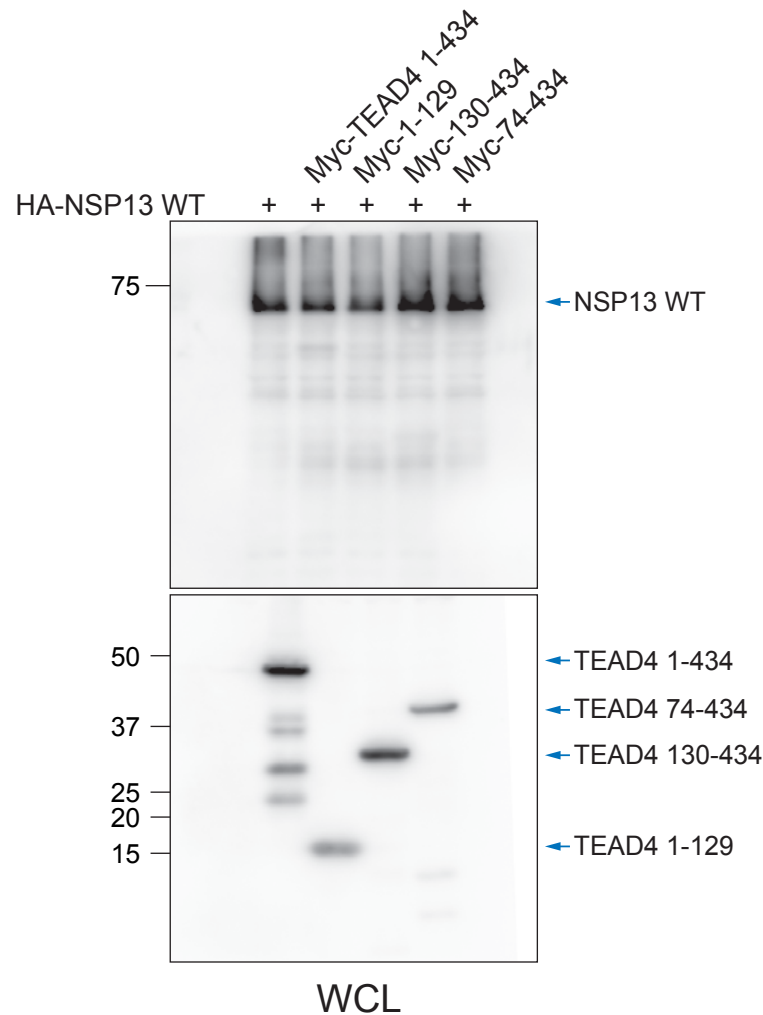

Supplement: Figure 4—figure supplement 2—source data 2. [file elife-100248-fig4-figsupp2-data2.zip › sFigure 4B.pdf]

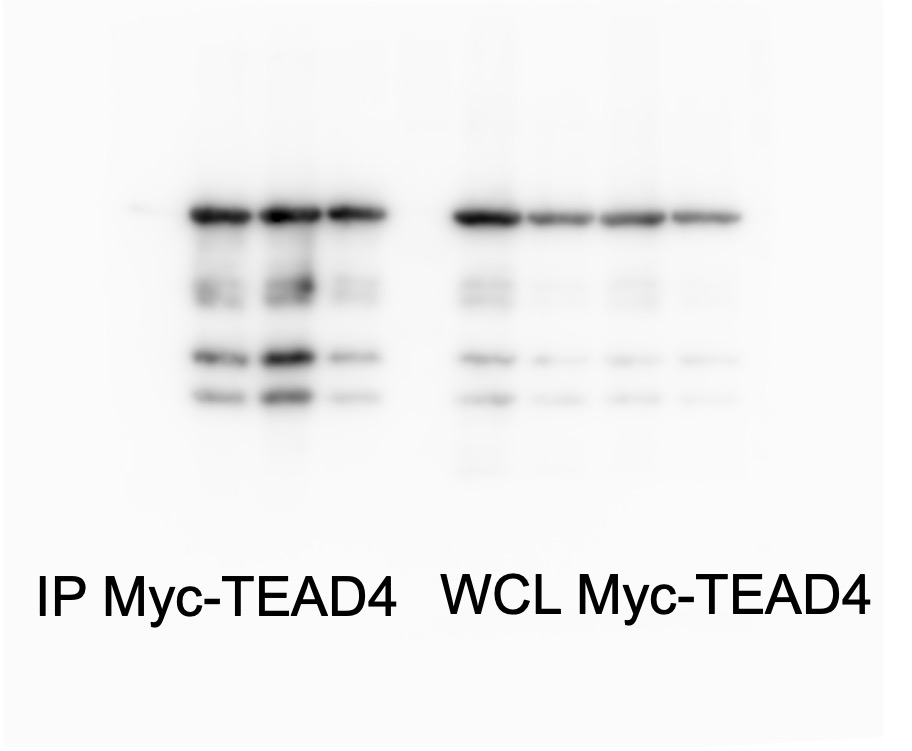

Supplement: Figure 4—figure supplement 3—source data 1. [file elife-100248-fig4-figsupp3-data1.zip › Myc-TEAD4 in IP and WCL.jpg]

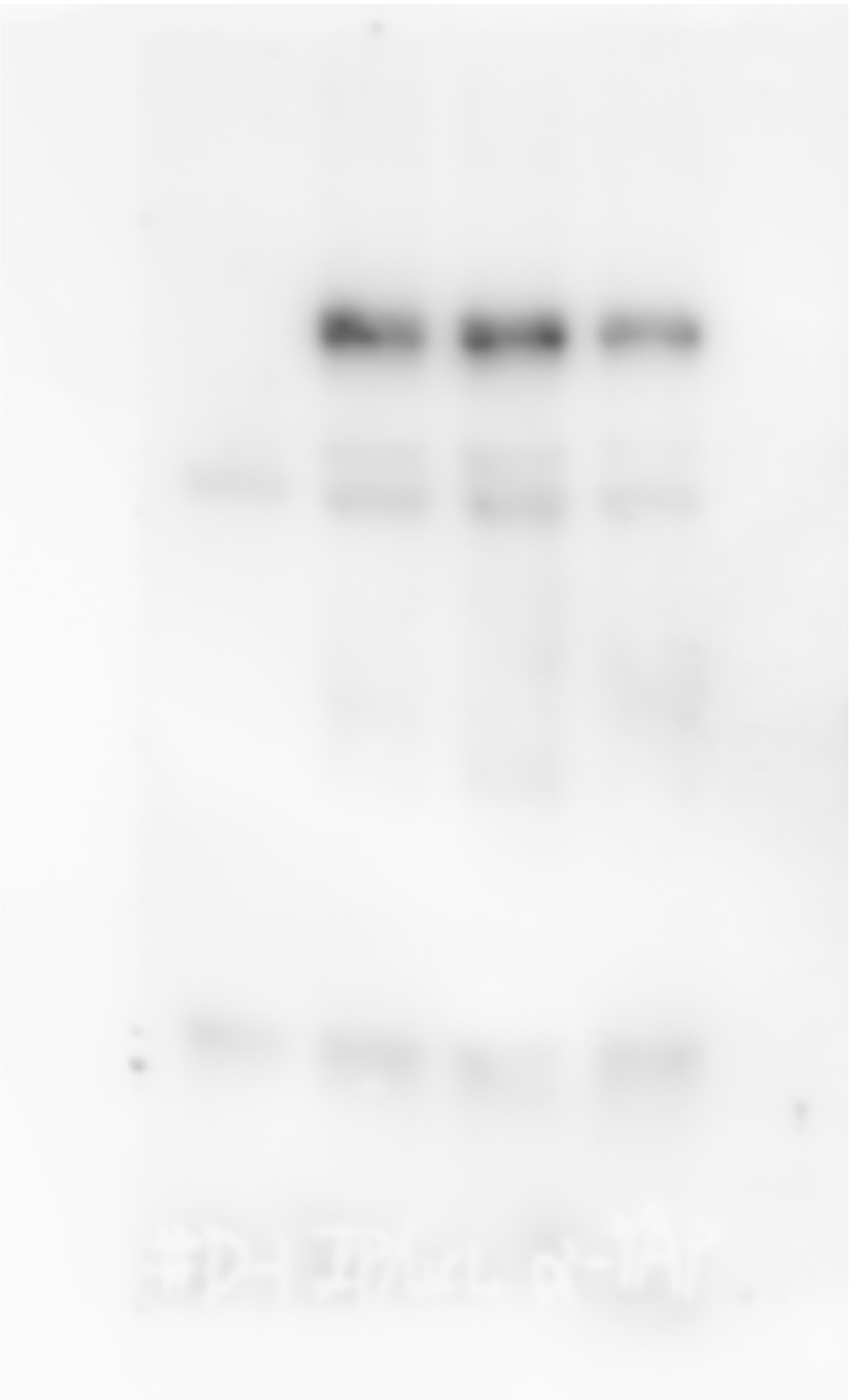

Supplement: Figure 4—figure supplement 3—source data 1. [file elife-100248-fig4-figsupp3-data1.zip › Flag-YAP in IP.jpg]

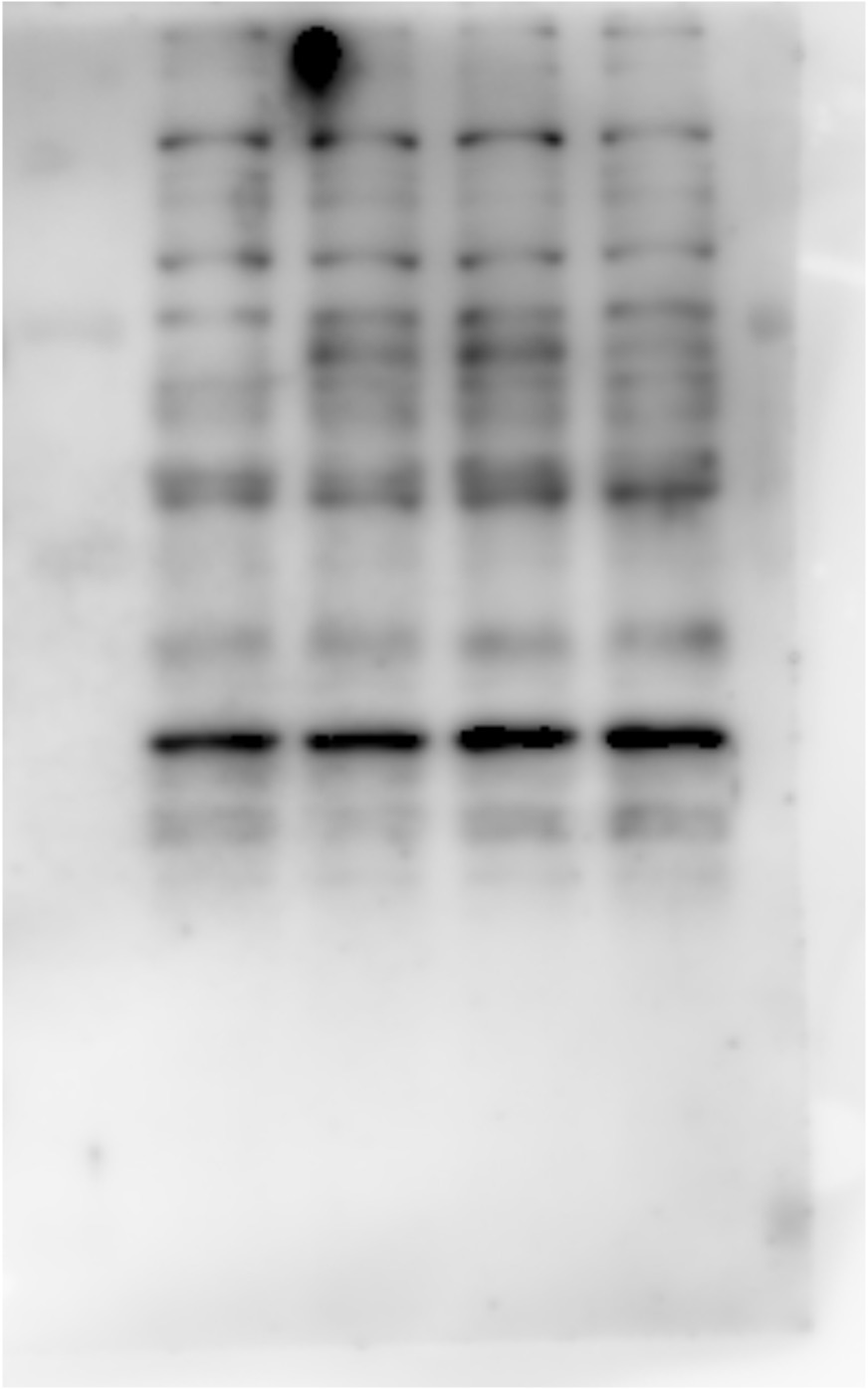

Supplement: Figure 4—figure supplement 3—source data 1. [file elife-100248-fig4-figsupp3-data1.zip › Flag-YAP in WCL.jpg]

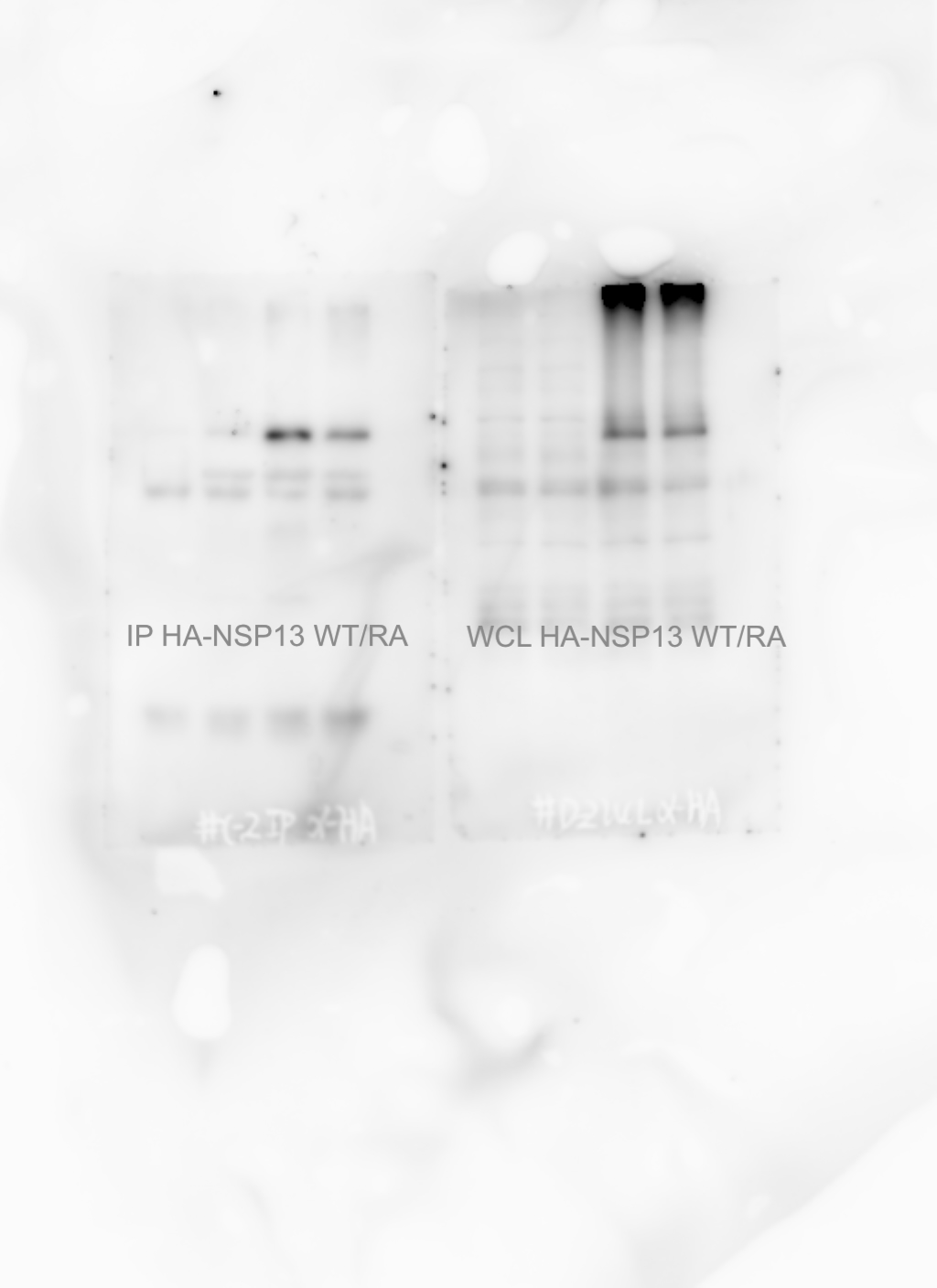

Supplement: Figure 4—figure supplement 3—source data 1. [file elife-100248-fig4-figsupp3-data1.zip › HA-NSP13 in IP and WCL.jpg]

sFigure 4C

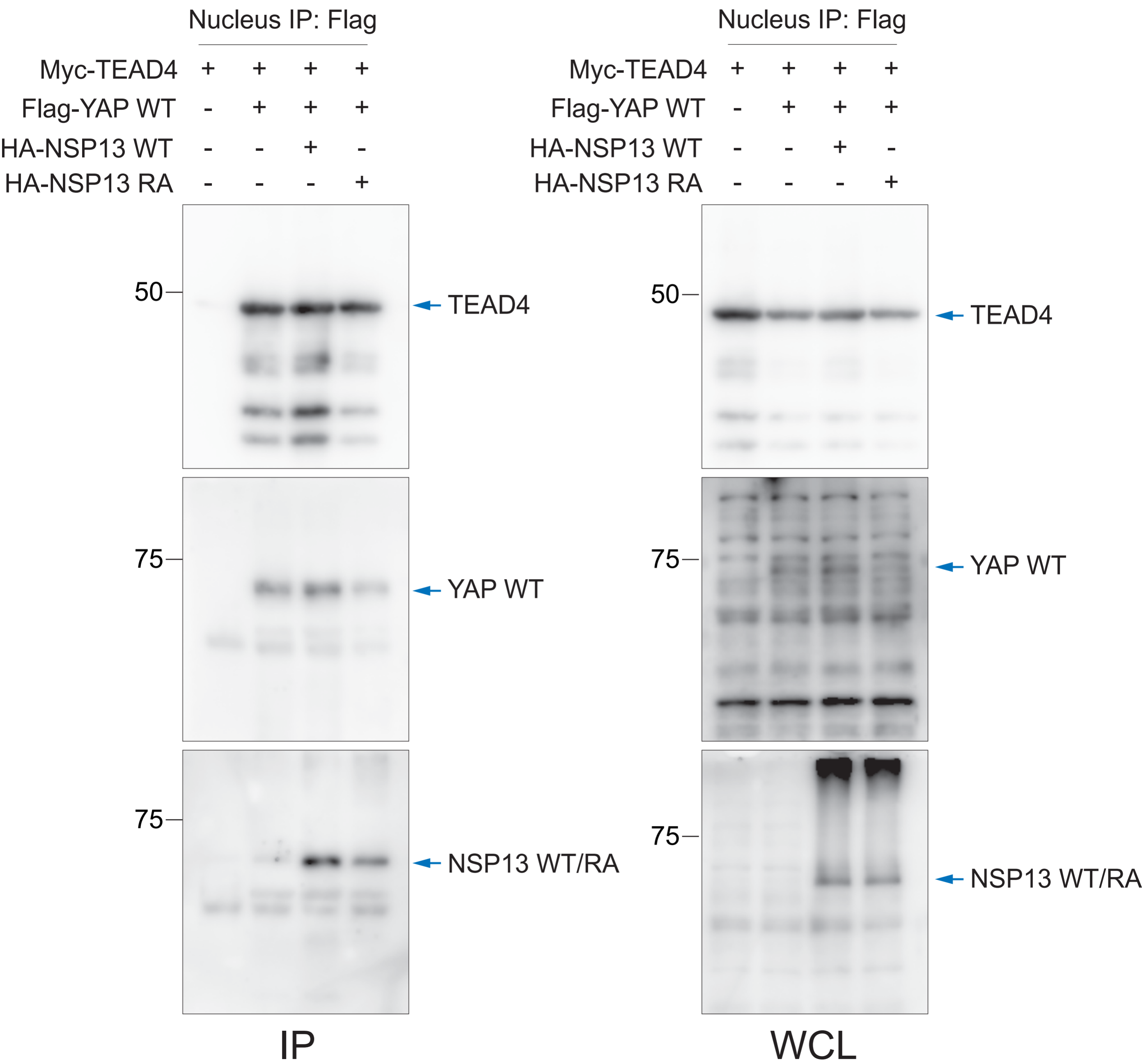

Supplement: Figure 4—figure supplement 3—source data 2. [file elife-100248-fig4-figsupp3-data2.zip › sFigure 4C.pdf]

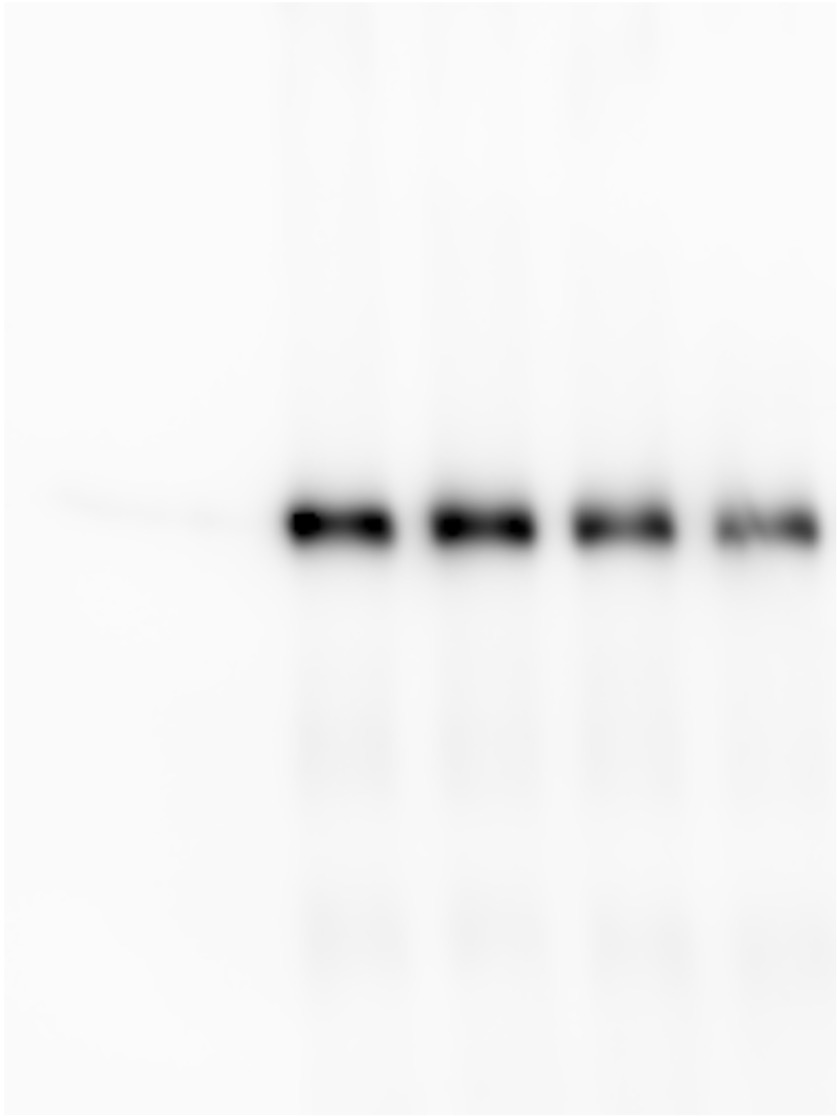

Supplement: Figure 4—figure supplement 5—source data 1. [file elife-100248-fig4-figsupp5-data1.zip › HA-NSP13 in IP.jpg]

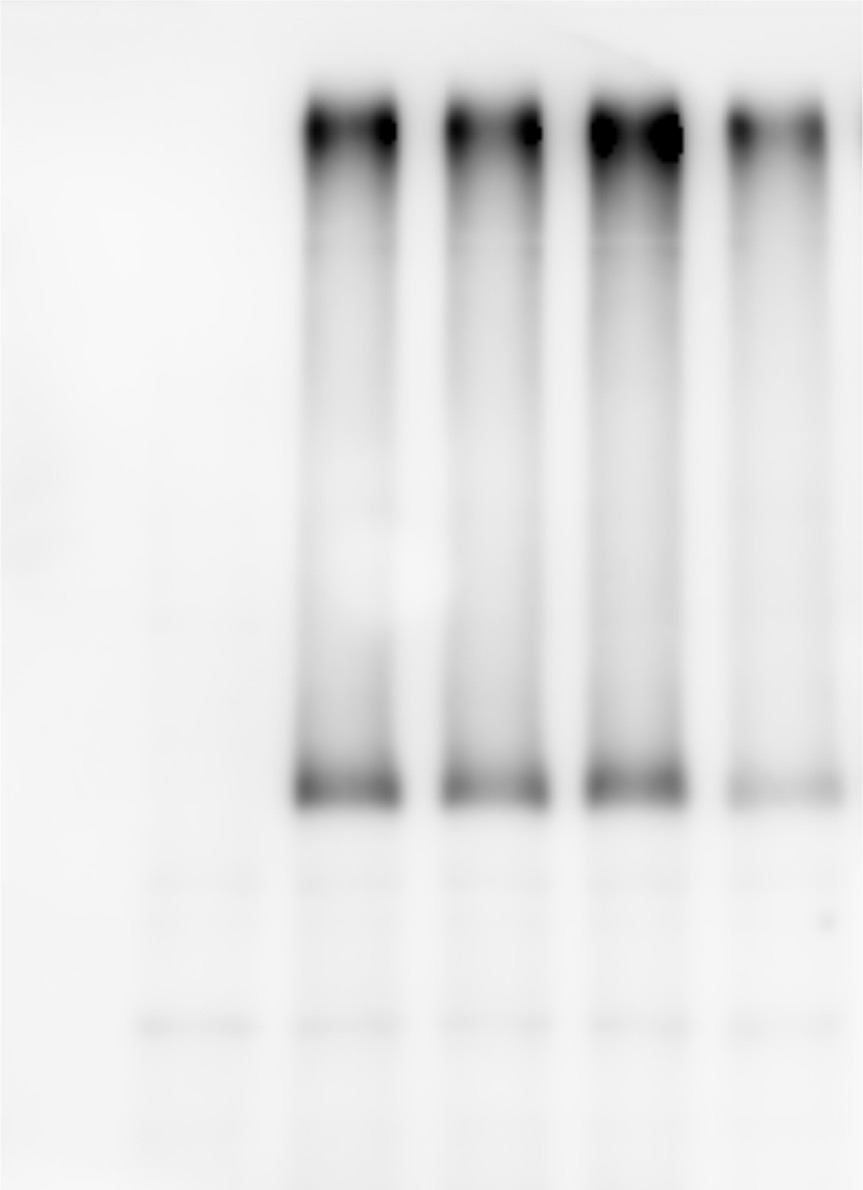

Supplement: Figure 4—figure supplement 5—source data 1. [file elife-100248-fig4-figsupp5-data1.zip › HA-NSP13 in WCL.jpg]

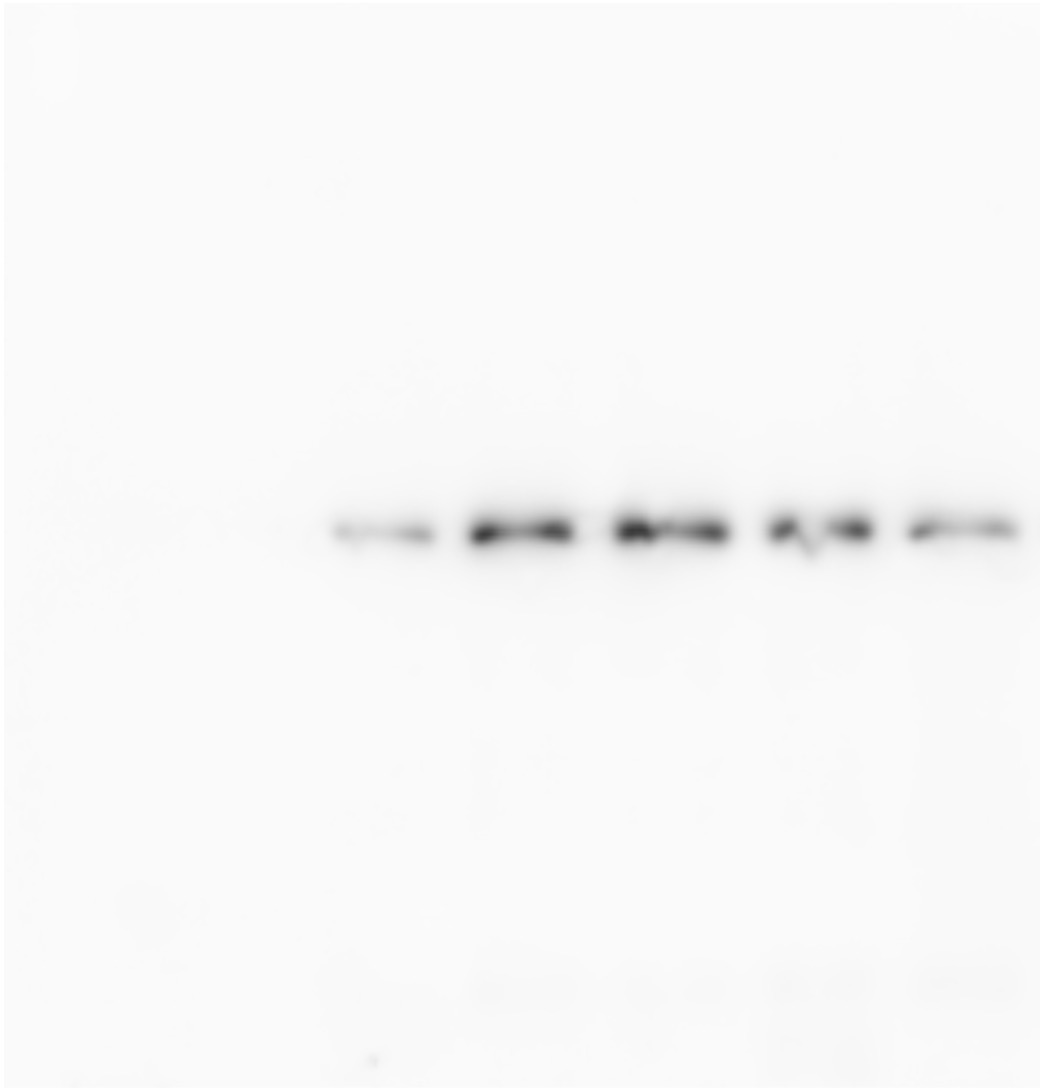

Supplement: Figure 4—figure supplement 5—source data 1. [file elife-100248-fig4-figsupp5-data1.zip › Myc-TEAD4 in IP.jpg]

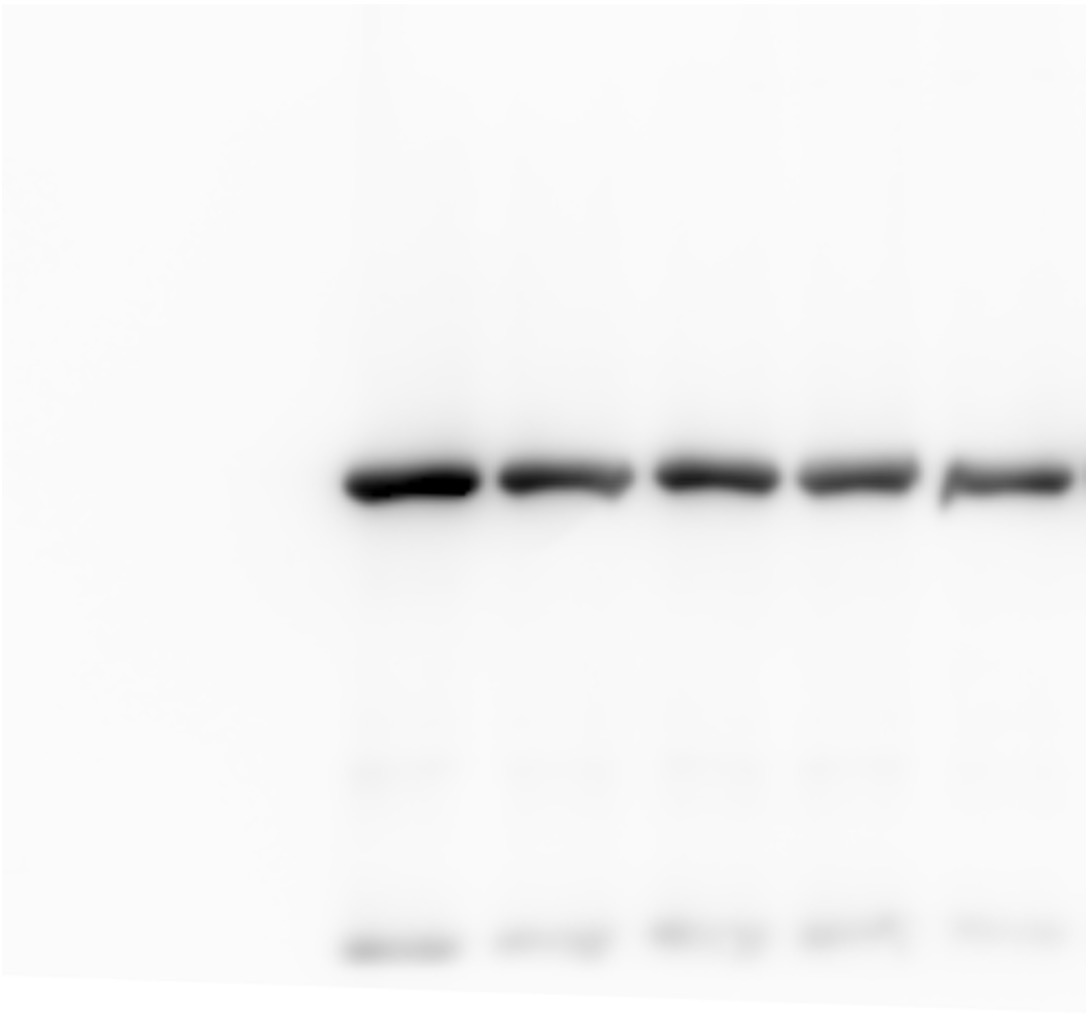

Supplement: Figure 4—figure supplement 5—source data 1. [file elife-100248-fig4-figsupp5-data1.zip › Myc-TEAD4 in WCL.jpg]

sFigure 4E

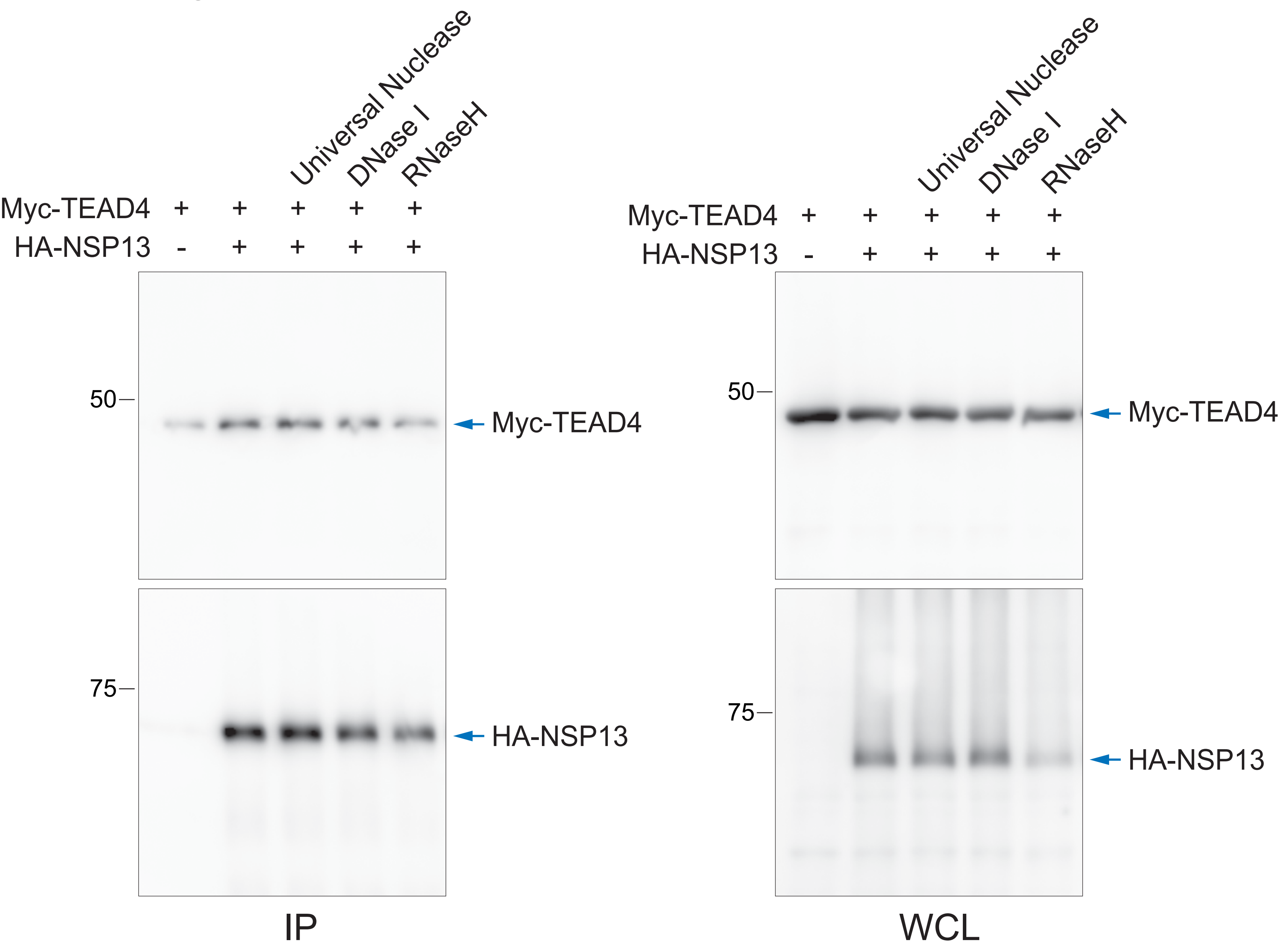

Supplement: Figure 4—figure supplement 5—source data 2. [file elife-100248-fig4-figsupp5-data2.zip › sFigure 4E.pdf]

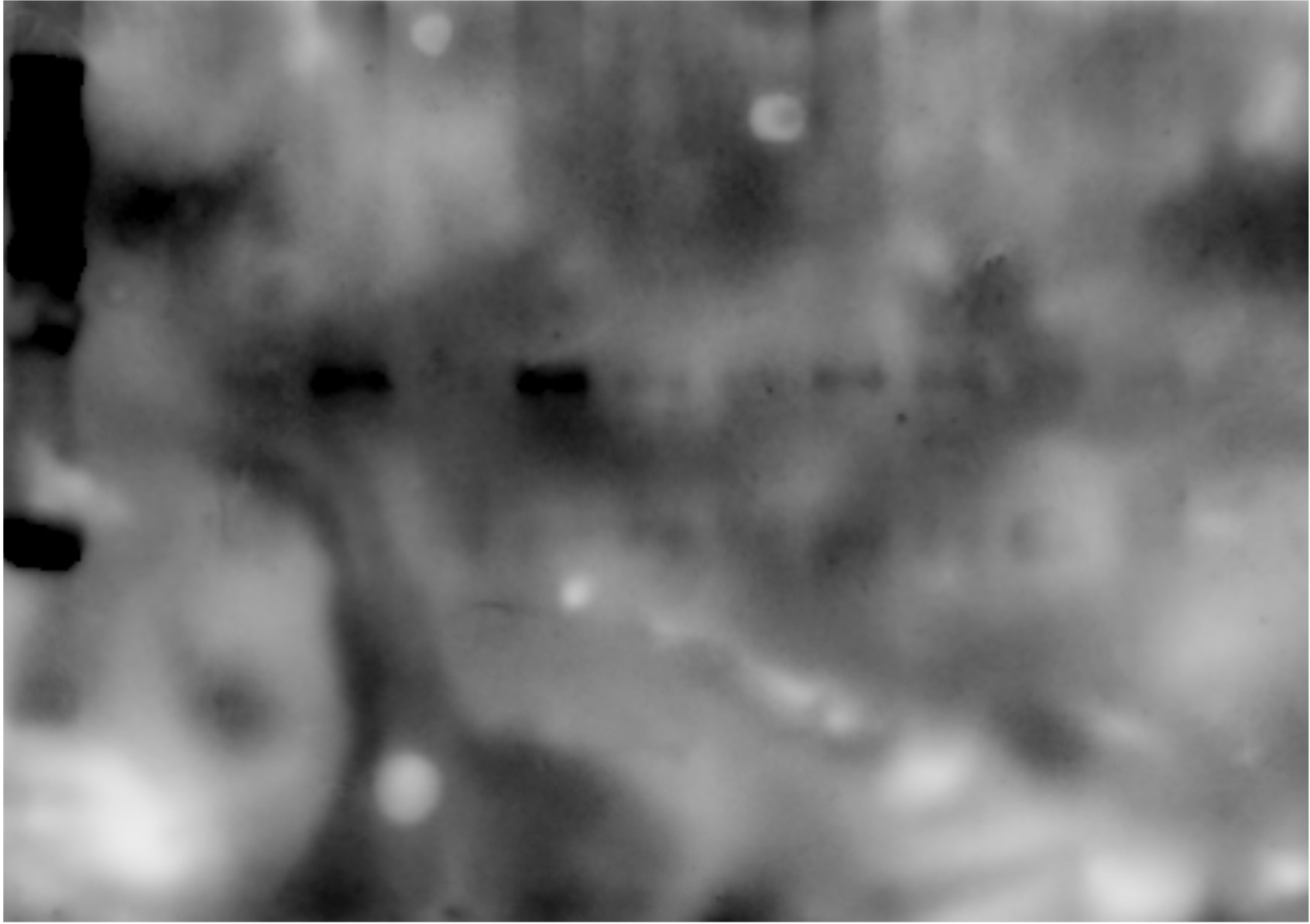

Supplement: Figure 4—figure supplement 7—source data 1. [file elife-100248-fig4-figsupp7-data1.zip › HA-NSP13 in IP long exposure.jpg]

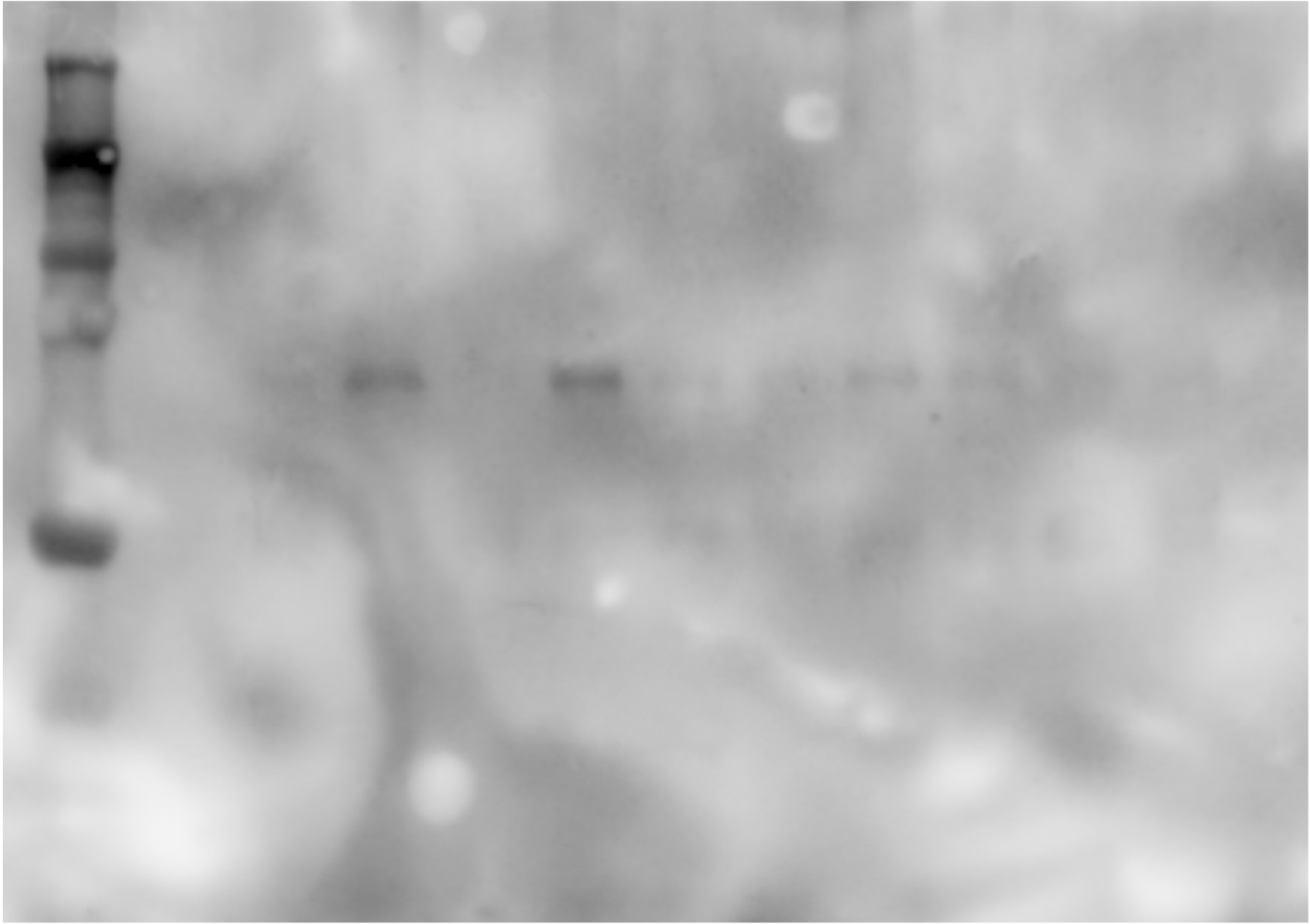

Supplement: Figure 4—figure supplement 7—source data 1. [file elife-100248-fig4-figsupp7-data1.zip › HA-NSP13 in IP.jpg]

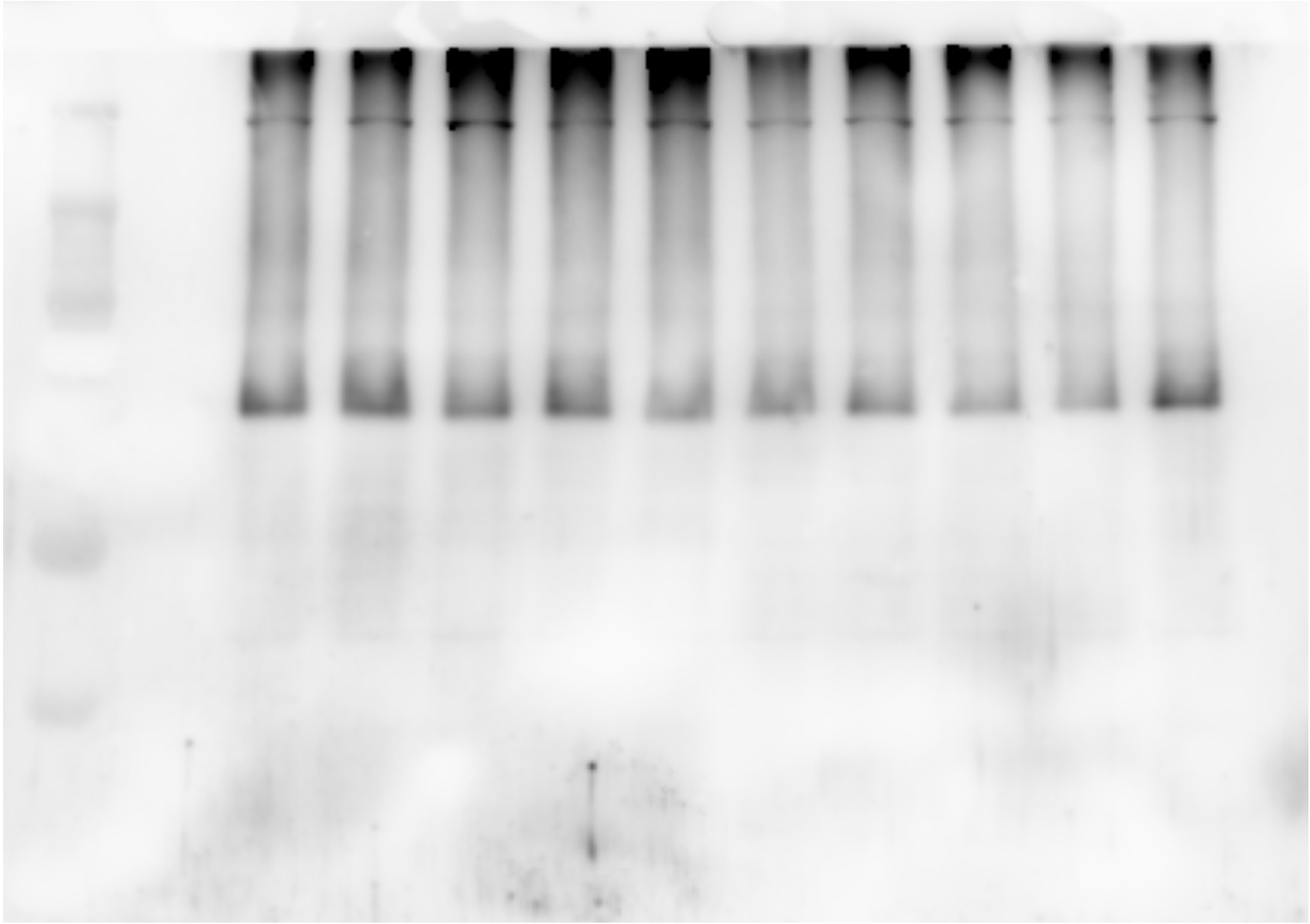

Supplement: Figure 4—figure supplement 7—source data 1. [file elife-100248-fig4-figsupp7-data1.zip › HA-NSP13 in WCL.jpg]

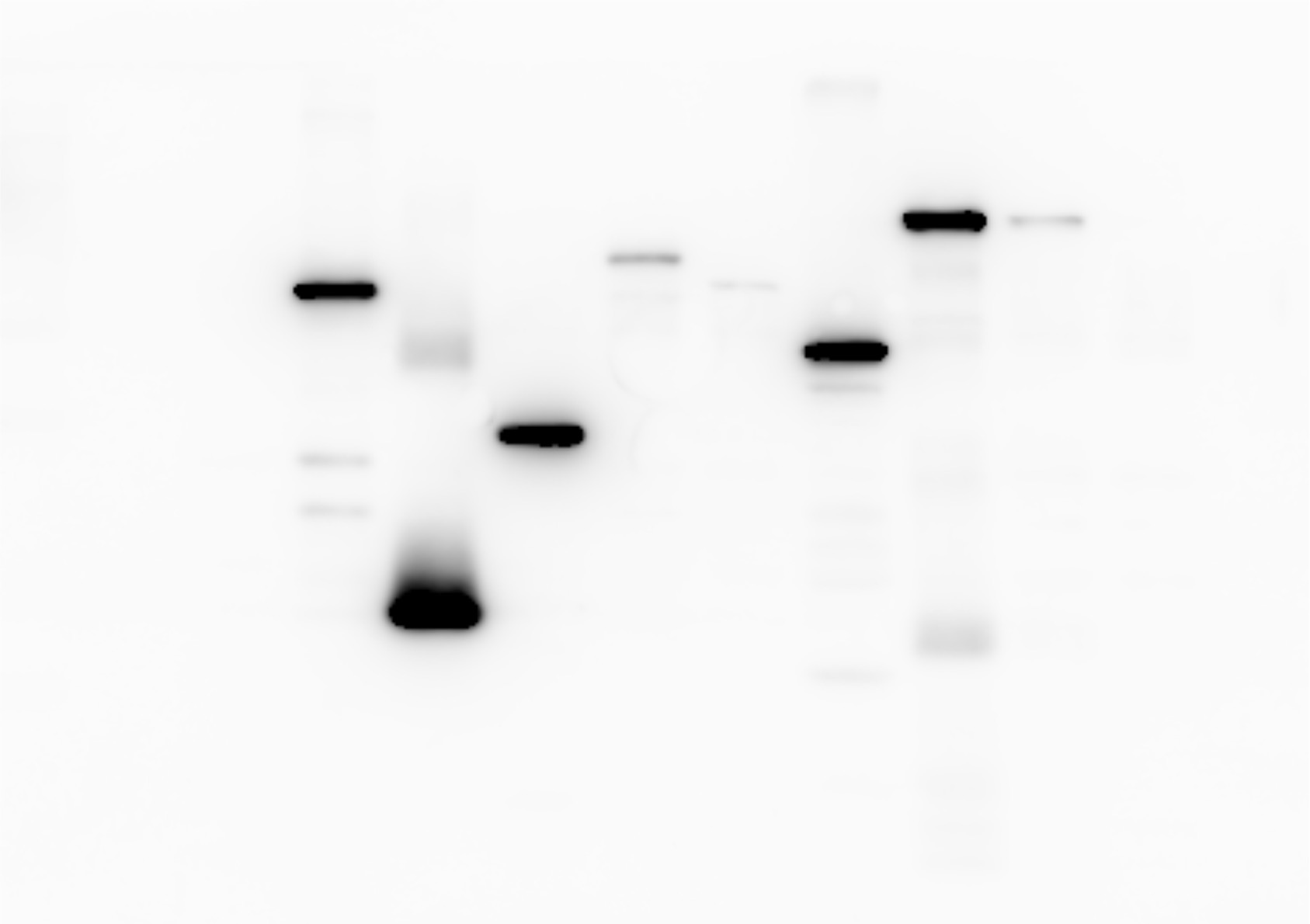

Supplement: Figure 4—figure supplement 7—source data 1. [file elife-100248-fig4-figsupp7-data1.zip › Myc tagged proteins in IP.jpg]

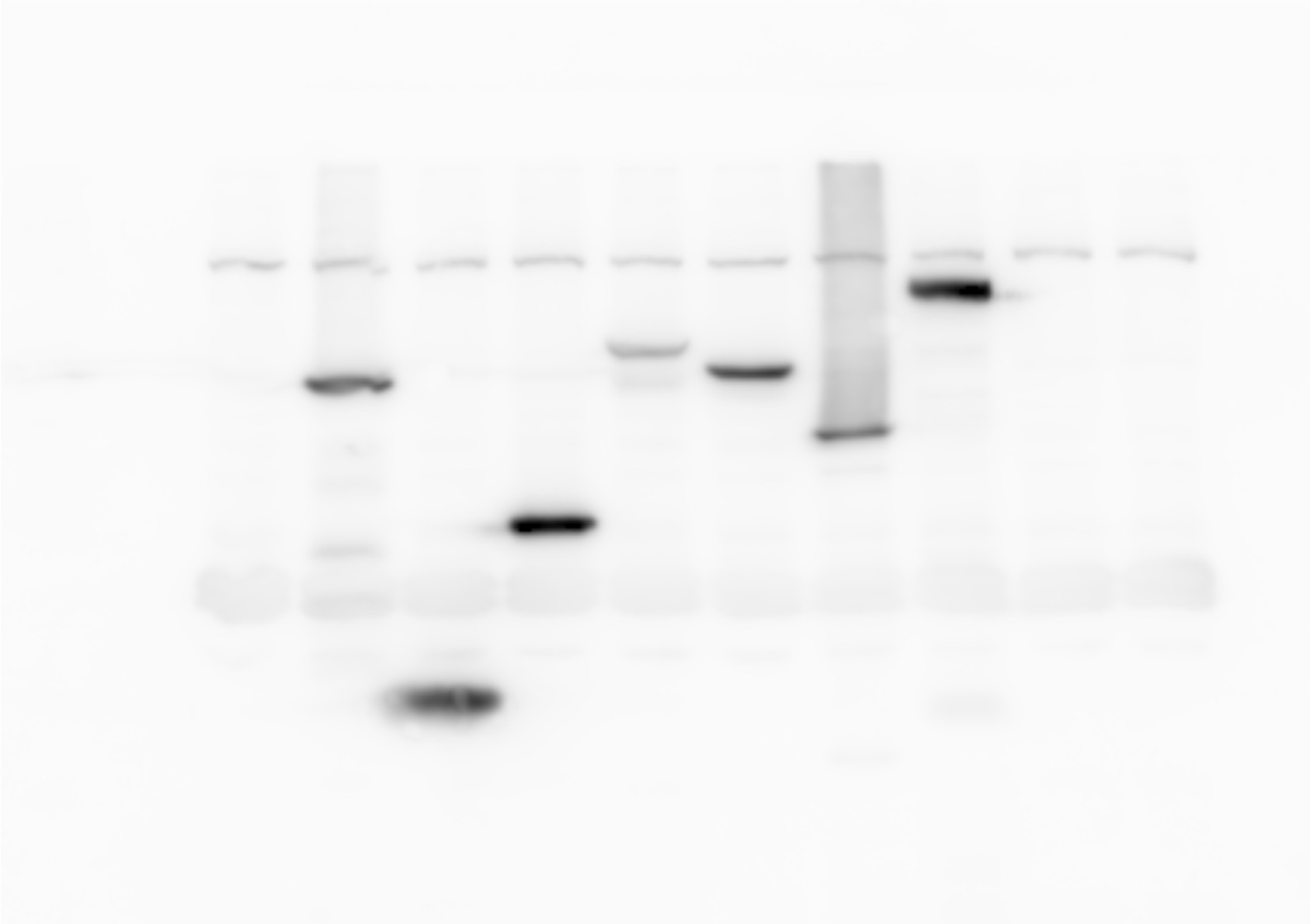

Supplement: Figure 4—figure supplement 7—source data 1. [file elife-100248-fig4-figsupp7-data1.zip › Myc tagged proteins in WCL.jpg]
