# Supplementary material for: SARS-CoV-2 NSP13 interacts with TEAD to suppress Hippo-YAP signaling
Source: eLife. 2025 Sep 23;13:RP100248. doi: 10.7554/eLife.100248 (PMC12456957; doi:10.7554/eLife.100248)
Supplement: Figure 4—source data 4. [file elife-100248-fig4-data4.zip › Figure 4C.pdf]

Figure 4C

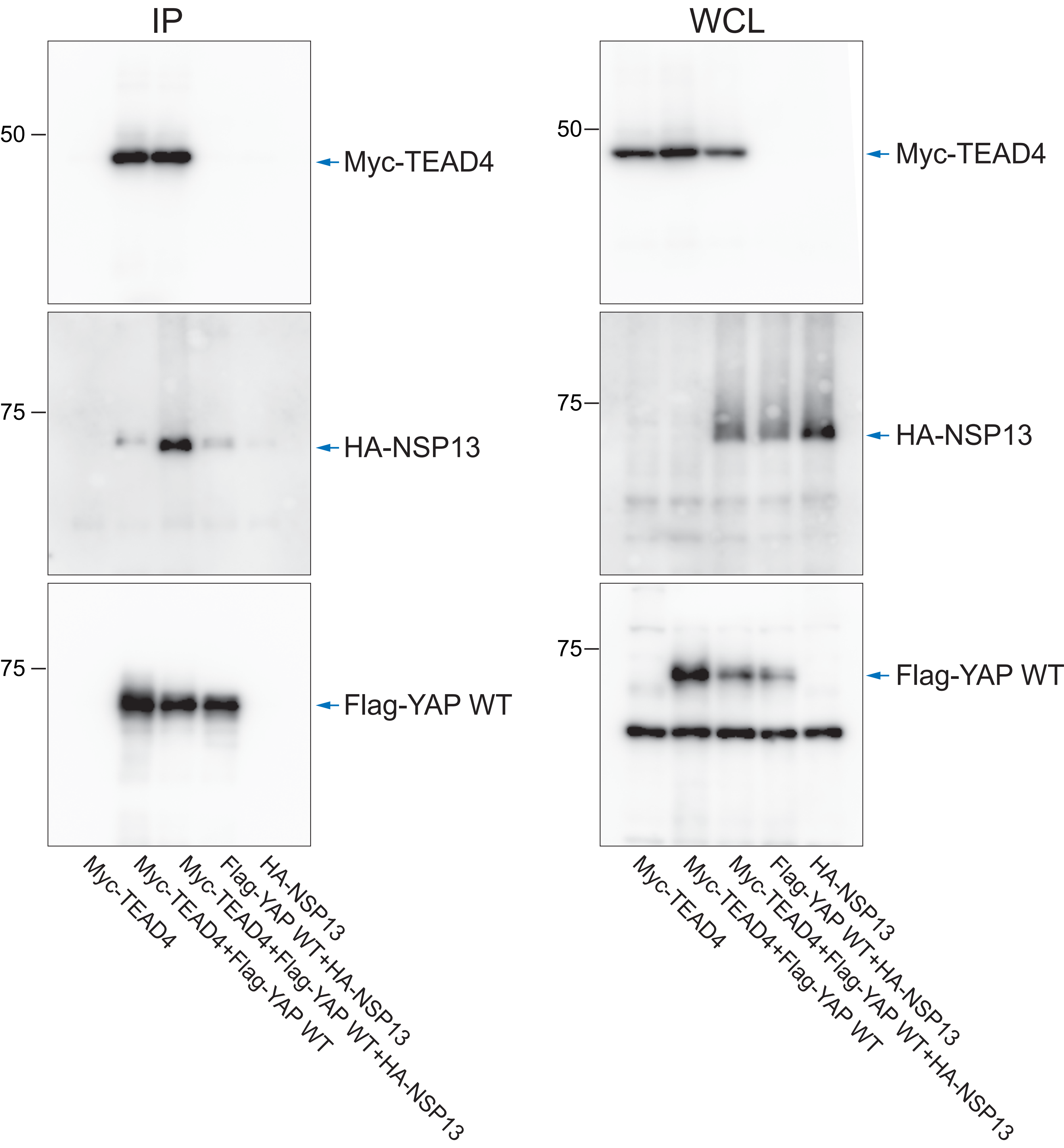

IP
WCL
50
75
75
50
75
75
Myc-TEAD4
HA-NSP13
Flag-YAP WT
Myc-TEAD4
HA-NSP13
Flag-YAP WT
Myc-TEAD4
Myc-TEAD4+Flag-YAP WT
Myc-TEAD4+Flag-YAP WT+HA-NSP13
Flag-YAP WT+HA-NSP13
HA-NSP13
Myc-TEAD4
Myc-TEAD4+Flag-YAP WT
Myc-TEAD4+Flag-YAP WT+HA-NSP13
Flag-YAP WT+HA-NSP13
HA-NSP13
