# Supplementary material for: SARS-CoV-2 NSP13 interacts with TEAD to suppress Hippo-YAP signaling
Source: eLife. 2025 Sep 23;13:RP100248. doi: 10.7554/eLife.100248 (PMC12456957; doi:10.7554/eLife.100248)
Supplement: Figure 4—figure supplement 7—source data 2. [file elife-100248-fig4-figsupp7-data2.zip › sFigure 5B.pdf]

sFigure 5B

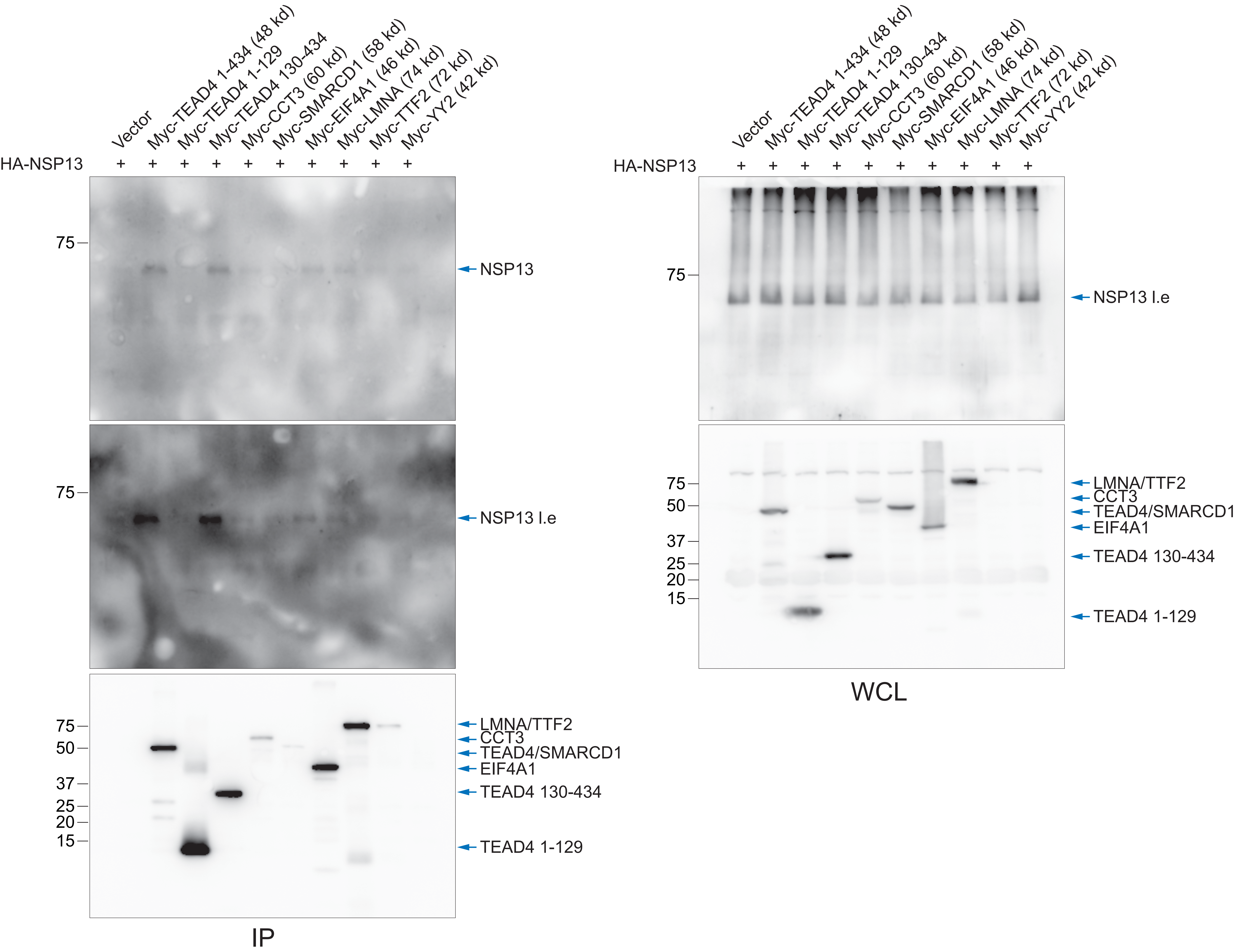

Vector
Myc-TEAD4 1-434 (48 kd)
Myc-TEAD4 1-129
Myc-TEAD4 130-434
Myc-CCT3 (60 kd)
Myc-SMARCD1 (58 kd)
Myc-EIF4A1 (46 kd)
Myc-LMNA (74 kd)
Myc-TTF2 (72 kd)
Myc-YY2 (42 kd)
HA-NSP13
+ + + + + + + + + +
75
NSP13
75
NSP13 l.e
75
50
37
25
20
15
LMNA/TTF2
CCT3
TEAD4/SMARCD1
EIF4A1
TEAD4 130-434
TEAD4 1-129
IP
Vector
Myc-TEAD4 1-434 (48 kd)
Myc-TEAD4 1-129
Myc-TEAD4 130-434
Myc-CCT3 (60 kd)
Myc-SMARCD1 (58 kd)
Myc-EIF4A1 (46 kd)
Myc-LMNA (74 kd)
Myc-TTF2 (72 kd)
Myc-YY2 (42 kd)
HA-NSP13
+ + + + + + + + + +
75
NSP13 l.e
75
50
37
25
20
15
LMNA/TTF2
CCT3
TEAD4/SMARCD1
EIF4A1
TEAD4 130-434
TEAD4 1-129
WCL
